# FreiBox: A Versatile Open-Source Behavioral Setup for Investigating the Neuronal Correlates of Behavioral Flexibility via 1-Photon Imaging in Freely Moving Mice

**DOI:** 10.1523/ENEURO.0469-22.2023

**Published:** 2023-04-25

**Authors:** Brice De La Crompe, Megan Schneck, Florian Steenbergen, Artur Schneider, Ilka Diester

**Affiliations:** 1 Optophysiology–Optogenetics and Neurophysiology, University of Freiburg, 79110 Freiburg, Germany; 2 Institute of Biology III, Faculty of Biology, University of Freiburg, 79104 Freiburg, Germany; 3 Intelligent Machine-Brain Interfacing Technology (IMBIT)-BrainLinks-BrainTools, University of Freiburg, 79110 Freiburg, Germany; 4 Bernstein Center for Computational Neuroscience, University of Freiburg, 79104 Freiburg, Germany

**Keywords:** 1-photon imaging, behavioral flexibility, Miniscope V4, open-source

## Abstract

To survive in a complex and changing environment, animals must adapt their behavior. This ability is called behavioral flexibility and is classically evaluated by a reversal learning paradigm. During such a paradigm, the animals adapt their behavior according to a change of the reward contingencies. To study these complex cognitive functions (from outcome evaluation to motor adaptation), we developed a versatile, low-cost, open-source platform, allowing us to investigate the neuronal correlates of behavioral flexibility with 1-photon calcium imaging. This platform consists of FreiBox, a novel low-cost Arduino behavioral setup, as well as further open-source tools, which we developed and integrated into our framework. FreiBox is controlled by a custom Python interface and integrates a new licking sensor (strain gauge lickometer) for controlling spatial licking behavioral tasks. In addition to allowing both discriminative and serial reversal learning, the Arduino can track mouse licking behavior in real time to control task events in a submillisecond timescale. To complete our setup, we also developed and validated an affordable commutator, which is crucial for recording calcium imaging with the Miniscope V4 in freely moving mice. Further, we demonstrated that FreiBox can be associated with 1-photon imaging and other open-source initiatives (e.g., Open Ephys) to form a versatile platform for exploring the neuronal substrates of licking-based behavioral flexibility in mice. The combination of the FreiBox behavioral setup and our low-cost commutator represents a highly competitive and complementary addition to the recently emerging battery of open-source initiatives.

## Significance Statement

Behavioral flexibility is essential to survive in a complex and changing environment. To study this cognitive ability in freely moving mice, we developed a versatile, low-cost, open-source behavioral setup, called FreiBox, allowing us to investigate the neuronal correlates of licking-based behavioral flexibility. FreiBox is controlled by a custom Python interface and integrates a new licking sensor for controlling spatial licking behavioral tasks (e.g., discriminative learning, reversal learning). We also developed and validated an active commutator to record calcium imaging with the Miniscope V4 in freely moving mice. Finally, we demonstrated that FreiBox can be associated with 1-photon imaging and other open-source initiatives to form a versatile platform for exploring the neuronal substrates of licking-based behavioral flexibility in mice.

## Introduction

Behavioral flexibility is essential to live in a complex and changing environment and to adapt to the infinite contexts of our daily life. An action that was appropriate in the past can become obsolete after a contextual change. This cognitive ability requires multiple mental processes which are supported by a vast cortico-subcortical network (Schoenbaum et al., 2009; Floresco and Jentsch, 2011; Izquierdo et al., 2017). By using behavioral flexibility paradigms, it is possible to explore cortical, basal ganglia, or thalamic network activities, as well as to cover many fields in behavioral neuroscience (e.g., sensory discrimination, decision-making). To do so in an animal model, we need to measure behavioral responses that can be attributed to association, contingency learning, decision, or adaptation.

**Figure 1.**
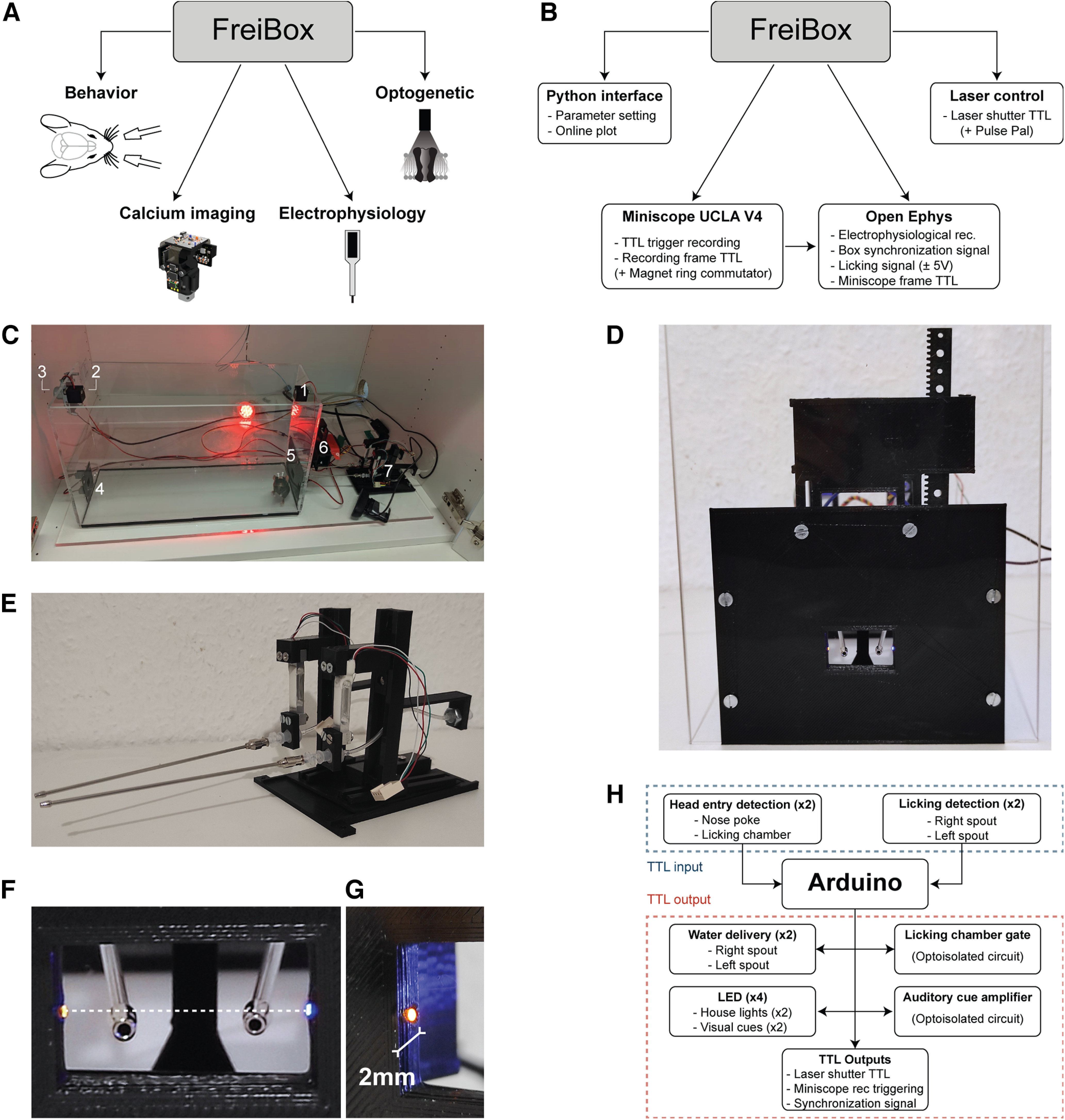
FreiBox platform and its operant instruments. ***A***, The FreiBox platform combines licking-based behavior with 1-photon calcium imaging, electrophysiological recordings, and optogenetic manipulation. ***B***, FreiBox is connected to a workstation equipped with a Python interface to set the task parameters and perform online analyses of the behavioral results. To perform optogenetic experiments, FreiBox can control a laser directly with an Arduino (Laser shutter) or indirectly via a PulsePal device. An Open Ephys acquisition system integrated into the platform performs electrophysiological recordings and can be used to synchronize behavioral events (such as the box synchronization and the licking signal) and the frame signal of the Miniscope UCLA V4 register. An open-source and cost-efficient commutator has also been developed to perform calcium imaging (see Extended Data Fig. 4-1). See Table 1 for the cost estimation of the FreiBox platform. ***C***, Pictures of the different components assembled to build FreiBox. Component numbers: 1 and 2, house light; 3, speaker; 4, NP chamber; 5, LC; 6, LC Gate; 7, SG lickometer. ***D–G***, Picture of the licking chamber (***D***) equipped with a gate, two lickometers (***E***), and an HE detection module, based on the breaking of an IR beam (***F***) relayed by two fiber optics placed at 2 mm from the entrance (***G***). ***H***, Input/output TTL signal mapping received and sent by the Arduino to react and control the electronic modules. In response to an HE in the NP/LC or licking on the spouts, the Arduino sends a TTL pulse to deliver water, turn on and off the house lights, open the LC gate, generate an auditory cue, generate a synchronization signal, and control other devices (e.g., a laser shutter). See Extended Data Figure 1-1 for the evaluation of the input/output reactivity of FreiBox and Extended Data Figure 1-2 for the electronic circuits of the FreiBox modules.

Typical behavioral responses in freely moving animals are nose pokes (NPs) in specific chambers, visits in maze arms, presses on a specific lever, or movements of a joystick in a specific direction to obtain a reward (Castañé et al., 2010; Parker et al., 2016; Izquierdo et al., 2017; Belsey et al., 2020; Lee et al., 2020; Mazziotti et al., 2020; Vassilev et al., 2022). For instance, Vassiliev et al. (2022) recently developed a well documented and open-source setup to perform a Go/No-Go task. While this approach is already very useful to explore behavioral flexibility in freely moving mice, no currently available setup permits measurement of directional licking, a behavioral paradigm that can access numerous cognitive processes in head-restrained mice (Mayrhofer et al., 2019; Wu et al., 2020; Bollu et al., 2021; Catanese and Jaeger, 2021; Wang et al., 2021). Such a tool would provide the opportunity to perform translational studies between head-fixed and freely moving conditions. Focusing on licking behavior, we have designed a fully open-source and cost-efficient (∼900 €, Table 1) behavioral platform to explore behavioral flexibility in freely moving mice. This new box, called FreiBox (Fig. 1), is capable of monitoring a wide variety of licking-based tasks, such as discriminative learning (DL) and serial reversal learning (RL), by tracking online licks with strain gauge (SG)-based force lickometers.

**Figure 2.**
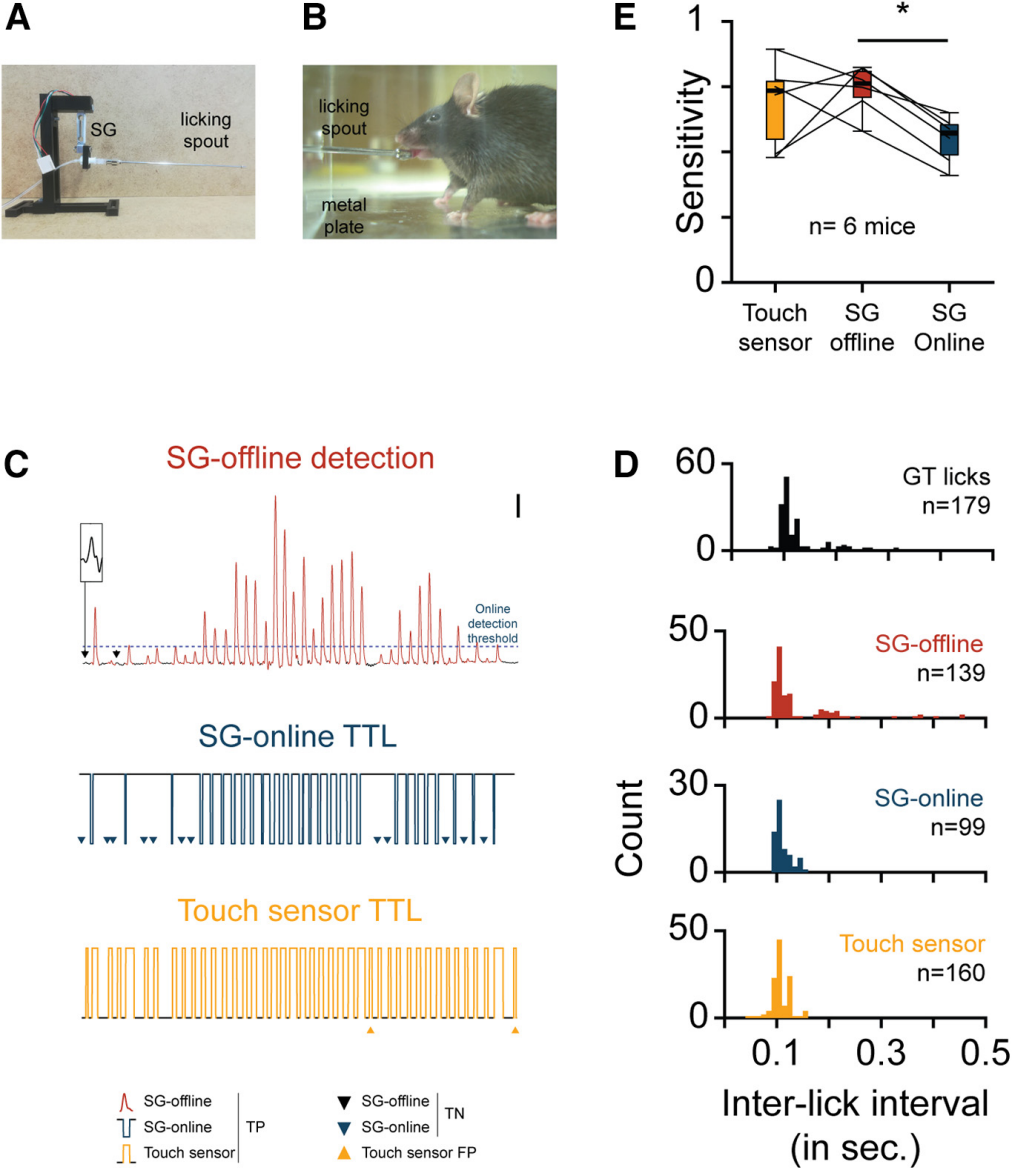
Validation of the strain gauge lickometer. ***A***, Picture of the SG lickometer, composed by a 3D-printed holder attaching an SG connected to a licking spout. ***B***, Picture taken by the high-speed camera used to define the ground truth licks and compare the sensitivity of the SG and touching lickometers. The metal plate on the box floor was used to ground the mice when the animal tongue touches the metallic spout. ***C***, 5 s example of TP, FN, and FP licks from the 3 licking detection methods (SG-offline, SG-online, and touch sensor), defined by comparison with the ground truth licks defined by a high-speed video recording. On the top trace (calibration: 5 s, 100 mV; inset calibration: 200 ms, 50 mV), the online detection threshold used to induce the SG-online TTL is plotted. ***D***, Distribution histogram of the interlick interval of the video GT and true-positive licks recorded with the 3 licking methods shown in ***C***. **(*E*).** Box-and-whisker plots comparing the sensitivity of touching sensor and SG lickometers. One-way repeated-measures ANOVA (*F* = 4.906, *p *=* *0.033) followed by Tukey’s test (*q* = 34.3, *p =* *0.031). See Extended Data Figure 2-1 describing the threshold method used to improve and monitoring the online lick detection.

10.1523/ENEURO.0469-22.2023.f1-1 Figure 1-1 Evaluation of the input/output reactivity of FreiBox. ***A***, ***B***, Measuring behavior requires integration and control of a myriad of different instruments in response to animal behavior by respecting a submillisecond timescale resolution. For example, the interlick interval is distributed at ∼100 ms (Fig. 2*D*), meaning that an Arduino controller has to integrate external information very quickly to measure animal behavior. To quantify the speed of FreiBox to integrate, respond, and time stamp such digital inputs, we conducted an experiment to measure the response reactivity (“I/O delay”) of an Arduino Mega to respond to an incoming TTL input. ***A***, Experimental setup and Arduino code used to measure the I/O delay of an Arduino Mega (“Output”) receiving an incoming TTL input (“Input”) driven by an experimenter controlling a 5V manual-push button. Arduino Mega was programmed to register the time stamp of the Input TTL (T1), detected with the Arduino library “DIO2,” and the time stamp of the TTL output (T2) right after its emission. In parallel, an oscilloscope (model DSO-1204e, Voltcraft) connected to a computer (DSO3104 software) recorded both input and output TTL to quantify the I/O delay at a fast sampling (500 kHz). ***B***, Example of the TTL traces recorded with an oscilloscope (sampling rate, 500 kHz). Calibration: 5 μs, 1 V. The oscilloscope I/O delay was obtained offline by measuring the delay between the input and output TTLs when their voltages were crossing 3.1 V (dashed line). ***C***, ***D***, In this experiment, we used 2 different boxes and mimicked 250 TTL inputs in each box by pressing a push button (*n* = 500 total trials). For these boxes, the I/O delays recorded with the Arduino (Box1 vs Box2: 6.03 × 10^–6^ ± 1.35 × 10^–7^ s vs 5.86 × 10^–6^ ± 1.28 × 10^–7^ s; *n* = 250 vs 250; Mann–Whitney rank-sum test: *t* = 64066.5, *p* = 0.359) and the oscilloscope (Box1 vs Box2: 5.90 × 10^–6^ ± 2.01 × 10^–7^ s vs 6.13 × 10^–6^ ± 1.94 × 10^–7^ s; *n* = 250 vs 250; Mann–Whitney rank-sum test: *t* = 61359, *p* = 0.433) were not statistically different. Hence, we pooled the data together. ***C***, Histogram distribution showing that the Arduino and oscilloscope I/O delays are not statistically different (“Arduino I/O delay” vs “Osci. I/O delay”: 6.01 × 10^−6^ ± 1.40 × 10^−7^ s vs 5.94 × 10^−6^ ± 9.30 × 10^−8^ s; *n* = 500; Friedman repeated-measures ANOVA on ranks: χ^2^ = 8 × 10^−3^, *p *=* *0.929). This result illustrates that an Arduino Mega 2560 can react to an incoming input with a delay of <20 μs. ***D***, Histogram distribution of the delay difference recorded simultaneously with the Arduino and the oscilloscope, showing that an Arduino can also register 2 close events with a precision of ±10 μs. Download Figure 1-1, TIF file.

10.1523/ENEURO.0469-22.2023.f1-2 Figure 1-2 Electronic circuits of the FreiBox modules. ***A***, ***B***, Amplification (***A***) and conditioning (***B***) circuit of the SG signal. The signal conditioning circuit is used to divide the voltage of the amplified signal (±12V) in a range that is acceptable for recording with most of DAC board (±5V). Note that the 100 Ω trimmer in ***A*** is used to adjust the sensitivity of the instrumental amplifier. ***C***, Voltage comparator circuit used to generate a TTL when the input voltage (*V*_in_) exceeds the value of a reference voltage (*V*_ref_). This circuit is used to detect both individual licks, and the IR beam breaking induced by a head entry in the NP or LC. The *V*_ref_ is controlled by a precise potentiometer (10 kΩ; tolerance, 5%; 10 turns) to fine-tune the licking detection or by a trimmer for head entry detection. ***D***, Electronic circuit of the head entry detection system. The generated signal (*V*_out_) is sent to the voltage comparator circuit described in ***C*** to generate TTL signal during a NP. ***E–H***, Electronic circuits of the Lighthouse LED Controller (***E***), water delivery system (***F***), the optoisolated gate controller (***G***), and the optoisolated auditory cure amplifier (***H***). Optoisolators are used to reduce the electrical noise induced by the motor in the FreiBox circuit and in the speaker. Download Figure 1-2, TIF file.

To track licking behavior online, conventional approaches use capacitive or optical methods to isolate individual licks. However, these methods generate electrical noise or are difficult to implement in freely moving mice. To address these issues, we developed a unique noiseless and sensitive mouse force lickometer ideally suited for freely moving conditions. To facilitate the integration of this new device into our platform, we added an adjustable online threshold detection system, making it compatible with nearly any behavioral platform, including those for head-fixed preparations. In addition, we designed a new licking chamber (LC) equipped with a gate, to control the accessibility of the licking spout, and a fiber optics-based infrared (IR) beam system with a very short sensing distance (2 mm) to facilitate its application to detect NP with implanted animals. We showed that by combining FreiBox with other open-source technologies, we are able to perform 1-photon calcium imaging in freely moving mice for a competitive cost per unit (<9000 €, Table 1). To this end, we have also included a low-cost (<150 €, Table 1) commutator that is equipped with a novel sensing system that improves online tracking of cable rotations without impairing the 1-photon calcium imaging via a Miniscope. Finally, to support the open-source philosophy, we built FreiBox by assembling multiple modules that are shared and fully available on the Github site of our laboratory (https://github.com/Optophys-Lab/FreiBox).

## Materials and Methods

### FreiBox platform and its operant instruments

Influenced by the lever-press tasks in which an animal chooses between pressing on the right or left lever (Boulougouris et al., 2007; Brady and Floresco, 2015), FreiBox (Fig. 1*C*,*D*) offers the mice directional licking. We equipped FreiBox with a NP and a LC placed at opposing sides of the Plexiglas box (40 × 20 × 15 cm; Fig. 1*C–H*). The LC contains two SG lickometers and a chamber gate (Fig. 1*E–G*). The LC gate provides control over the licking access, similar to the lever retraction in commercial boxes. Inspired by previous optical lickometers (Schoenbaum et al., 2001; Isett et al., 2018), we included an optic fiber IR detection system at 2 mm from the entrance of both chambers to detect head entries (Fig. 1*G*,*H*). This design offers the advantage of decreasing the detection distance from the entrance and reducing the risk for the animal to touch the box walls with its implants. In addition to the operant instruments, we added several components (Fig. 1*C*,*D*) that can be used to deliver water or to generate light or auditory cues. An UltiMaker 3D printer equipped with black PLA filament was used to make the 3D-printed parts.

To control FreiBox and its electronic modules, we used the programmable microcontroller Arduino Mega 2560 (Arduino; Fig. 1*I*). This board can be easily interfaced with a Python library (Pyserial) to control behavioral tasks, set the task parameters, or collect results to perform online plotting. Although this Arduino board has the major advantage of interacting directly with sensors or other electronic modules (e.g., sound card) through several analog and digital input/output (I/O) pins and multiple libraries (Fig. 1*I*), it is a “one core” device that can execute only one instruction at a time. This dramatically reduces the processing speed. According to the manufacturer (https://www.arduino.cc/reference/en/language/functions/analog-io/analogread/), the analog sampling rate is relatively slow (<10 kHz for a single channel) and decreases with the number of recorded channels. This contrasts strongly with the high-speed capability of an Arduino to read and write digital signals with the Arduino library “DIO2” (∼4 μs, 250 kHz; https://www.arduino.cc/reference/en/libraries/dio2/). The input/output reactivity of the Arduino Mega 2560 (Extended Data Fig. 1-1) has a delay of <20 μs. Based on those results, we built electrical modules which interact with the Arduino board by using digital signals only (Fig. 1*I*). This transistor–transistor logic (TTL)-based modular organization facilitates the electronic integration and transposition into different behavioral controller platforms (Raspberry Pi or Teensy). Hence, we developed several hardware modules that can be incorporated or removed depending on the task needs. For instance, since the Arduino Mega board has half of its digital pins available in FreiBox, it is possible to include several other spouts to perform sequence learning, without comprising the reading speed of more than several tens of microseconds per additional spout. We provide here the circuits used to build FreiBox (Extended Data Fig. 1-2).

### Sensitivity evaluation of the strain gauge lickometer

When a mouse licks on the spout of the SG lickometer (Fig. 2*A*,*B*), the amplified SG signal (SGs; Extended Data Fig. 1-2*A–C*) exhibits large peaks (Fig. 2*C*, Extended Data Fig. 2-1*A*) that can be easily recorded by any analog-to-digital converter (e.g., Open Ephys) and analyzed offline (see the following section) to extract individual licks (Fig. 2*C*, SG-offline detection). Given the large signal-to-noise ratio of the SGs, we connected a voltage comparator circuit (Extended Data Fig. 1-2*C*) to track the licking behavior online (Fig. 2*C*, SG-online TTL). Such a circuit generates a TTL pulse when the lick peaks of the SGs cross a defined reference voltage threshold. For the validation experiment described below, this threshold was set at 100 mV above the baseline and kept constant for all mice.

10.1523/ENEURO.0469-22.2023.f2-1 Figure 2-1 Improving online detection of the SG lickometer. ***A***, ***B***, A 5 s example of the SG raw signal (***A***; calibration, 0.1 V) and its online detection signal (***B***; SG-online TTL) recorded from another mouse (#JD1) than in Figure 2*C*. The SG-online TTL is generated when the SGs crosses an online reference voltage threshold set 100 mV above the baseline (“Online thres.”). By decreasing artificially this reference voltage (***A***, Artificial thresh.), we generated multiple artificial SG-TTL traces (***B***) to evaluate the effect of the voltage threshold on the sensitivity (***C***) and the PPV (***D***). ***C***, Box-and-whisker plots comparing the sensitivity of SG lickometer and of the artificial SG-TTL traces as described in ***B*** (*n* = 6; Friedman repeated-measures ANOVA on ranks: χ^2^ = 21.643, df = 7, *p *=* *0.003; TS vs SG-offline vs SG-online vs 0 mV vs 20 mV vs 40 mV vs 60 mV; 0.685 ± 0.0676 vs 0.740 ± 0.0374 vs 0.546 ± 0.0372 vs 0.549 ± 0.0413 vs 0.600 ± 0.0406 vs 0.626 ± 0.0486 vs 0.577 ± 0.121 vs 0.606 ± 0.126, respectively; describe data: mean ± SEM). *#Tukey’s *post hoc* test, *q* = 5, *p *<* *0.05. ***D***, Box-and-whisker plots comparing the PPV of SG lickometer and of the artificial SG-TTL traces as described in ***B*** (one-way repeated-measures ANOVA: *n* = 6, *F* = 5.523, *p *<* *0.001; TS vs SG-offline vs SG-online vs 0 mV vs 20 mV vs 40 mV vs 60 mV; 0.823 ± 0.0873 vs 0.813 ± 0.0785 vs 0.827 ± 0.0972 vs 0.729 ± 0.129 vs 0.684 ± 0.164 vs 0.621 ± 0.175 vs 0.517 ± 0.189 vs 0.402 ± 0.178, respectively; describe data: mean ± SEM. #Tukey’s *post hoc* test; 80 mV vs TS, 80 mV vs SG-Offline, 80 mV vs SG-Online, 80 mV vs 0 mV; *q* = 86.365, 86.205, 86.417, 84.941, respectively; *p *=* *0.002, 0.002, 0.002, 0.026 respectively. *Tukey’s *post hoc* test; 60 mV versus TS and 60 mV versus SG Online; *q* = 84.630 and 84.683, respectively; *p *=* *0.044 and 0.04, respectively. ***E***, Electronic circuit of the Arduino oscilloscope for online monitoring of the SG signals and their thresholds. Design modified from https://create.arduino.cc/projecthub/aimukhin/advanced-oscilloscope-955395. Download Figure 2-1, TIF file.

The term touch sensor (TS) refers to all methods and electronic circuits derived from the “Electronic Drinkometer” published by Hill and Stellar (1951). This device had been designed to detect a voltage or capacitive change when a rodent tongue touches the liquid; in other words, the animal becomes part of an electronic circuit (Schoenbaum et al., 2001) and “completes” it during licking (Hill and Stellar, 1951). To test whether the sensitivity of the SG lickometer (Fig. 2) is comparable to such classic TS, we placed six water-restricted mice in a water-delivery chamber (20 × 20 × 20 cm), containing a single licking spout attached to an SG (Fig. 2*A*) and connected to the TS circuit (Goltstein et al., 2018). The box floor was covered with a metal plate connected to the ground of the TS circuit. High-speed video recordings (1000 frames/s; Area Scan Camera, model ace acA1300-200uc, Basler) were simultaneously performed (Fig. 2*B*) to control the contact of the tongue of the mouse with the spout and measure the “ground truth licks” (GT-licks). The videos were then annotated offline with the software BORIS (Behavioral Observation Research Interactive Software) to extract the time stamps of the licks. To align the licking detection time stamps, the TTL from the camera (indicating the exposure window of each video frame) were recorded with an Open Ephys system (sampling rate, 30 kHz), simultaneously with the SGs, SG-online TTL, and TTL of the TS. The GT-licks were then aligned to the SGs (analog and SG-online TTL) and the TS TTL, to count true-positive (TP) and false-positive (FP) licks (Fig. 2*C*). The sensitivities (Sensitivity = [TP/(TP + FN)]; where FN is false-negative) and positive predictive values (PPVs; PPV = [TP/ (TP + FP)]) were evaluated to compare the detectors. The PPV was quantified to compare the false detection rate occurring with both lickometers. For the TS, FP licks occurred following an electrical artifact or when the mouse was in close contact or in immediate proximity to the spout or water droplet. For the SG, false detection occurred only when the SG signal crosses the reference voltage threshold of the comparator circuit, thus mainly when the mouse exerts a force pressure on the spout.

To extract individual licks, the SGs has been first downsampled (with an antialiasing low-pass filter) to 1 kHz (MATLAB function “resample”) and low-pass filtered at 64 Hz. The function “findpeak” (MATLAB) was then used to extract the peaks of individual licks. To improve the detection, we used the following parameters: a minimal peak distance of 60 ms, a minimal prominence of 0.015, and a minimal peak width of 50 ms. We applied a matching procedure to validate each detected lick, by comparing the delay of each detected lick with its closer GT value. A lick was considered as TP if this delay was less than the minimal interlick interval used for the SG-offline detection (60 ms).

### Animals and water restriction protocol

All animal procedures were performed in accordance with the guideline RL 2010/63/EU and approved by the Regierungspräsidium Freiburg. The animals were housed (Blueline type 1284 L, Tecniplast) with a humidity between 45% and 65%, and a temperature between 20°C and 24°C, under a 12 h light/dark cycle (light period from 8:00 A.M. to 8:00 P.M.). Mice received food *ad libitum*. A total of 11 mice (7–10 weeks of age, male) were used in this study: 8 C57BL/6J mice were used to validate the intrasession RL; 1 C57BL/6J and 2 Thy1-GCaMP6f (C57BL/6J-Tg(Thy1-GCaMP6f)GPS.5.17 DKim/J) mice were used to evaluate the frame loss percentage of the commutator and to explore the calcium activity of the orbitofrontal cortex (OFC) during DL.

To motivate the mice to perform the behavioral task, they were maintained under water restriction up to 5 d of the week followed by 1–2 d of water *ad libitum*. During the water restriction period, a careful monitoring of the weights of mice was performed on a daily basis. Care was taken to ensure that they did not weigh <80% of their free-feeding weight measured at the start of the week of deprivation. For training days, the animals consumed at least 1 ml of water. When weight fell to <80%, additional water was given until they recovered (>80% of the initial weight). In the periods without behavioral assays, the mice received water *ad libitum*.

### Behavioral task

**Figure 3.**
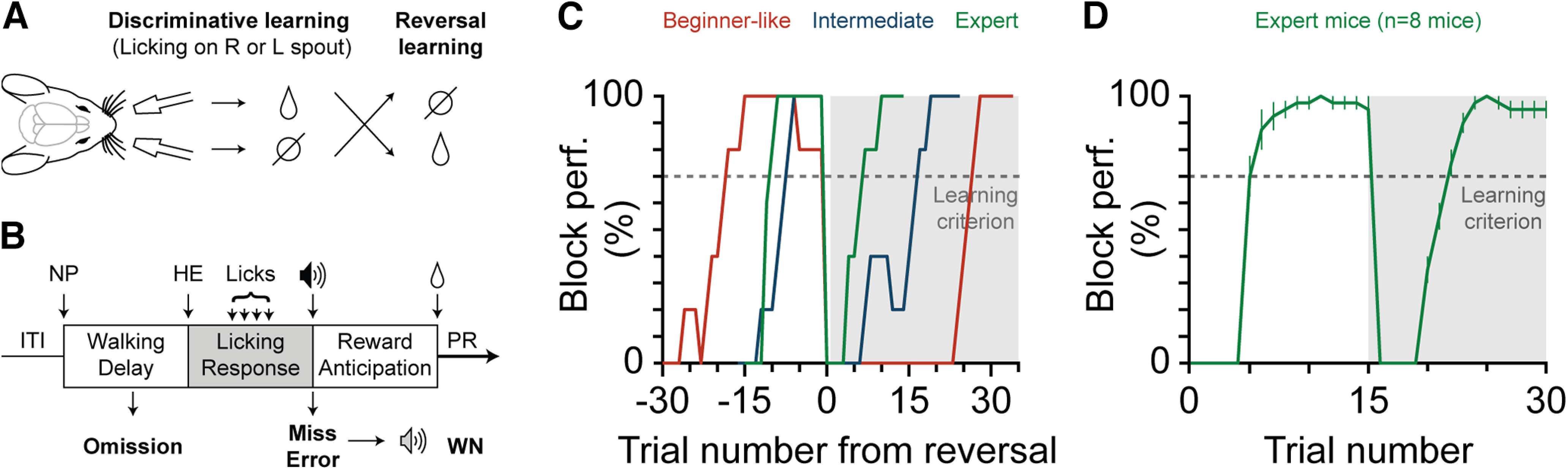
FreiBox control of intrasession reversal learning. ***A***, ***B***, Timeline (***A***) and description of the dual licking spouts intrasession RL task (***B***). ***C***, Typical session performance obtained from the same animal during three different sessions. An intermediate performance plot (blue, intermediate) has been selected to cover the performance range of the animal between its worst (red, beginner-like) and best (green, expert) sessions. ***D***, Population averaging (*n* = 8 mice) of the block performance during RL (mean ± SEM). For each expert animal, the best session was selected to perform averaging. The block performance was calculated with a moving average of 5 trials. See Extended Data Figure 3-1 showing individual behavioral data and the Python interface used for setting the behavioral parameters and control serial RL paradigm.

The RL task is a freely moving directional licking paradigm (Fig. 3*A–D*) adapted from a head-restraint condition (Mayrhofer et al., 2019). During the DL phase, the mice had to explore to find the reward spout by licking on one of the two available spouts (Fig. 3*A*). After the learning phase, the reward spout is automatically changed and the mice need to adapt their behavior (Fig. 3*A*, RL phase). To prevent to reverse the contingencies while they were not properly learned, the RL phase occurred only if the mouse reached (1) a minimum number of 15 trials (block length), and (2) an online performance criterion (70% hit in a sliding average window of 15 trials). To initiate a trial and to open the LC gate, the mice must perform a NP (Fig. 3*B*). They can then walk [walking delay (WD)] to enter their head into the LC [head entry (HE)] and lick on the correct spout during the licking response (LR) period (2 s). At the end of the LR, an auditory cue is played, consisting of a pure 5 kHz tone for the correct licking trials (or hit trials) or white noise for missed trials (entry in the LC without licking on a spout during the 2 s LR) or error trials (licking on the incorrect spout). After an incorrect or missed trial, the gate is automatically closed. In contrast, a reward anticipation (RA) period is maintained 1 s before the water delivery for the hit trials, to allow recording of reward-related activity prediction. In this configuration, the auditory cues become predictive of the outcomes. Finally, the LC gate is closed and the trial is finished after a 4 s postreward interval, allowing reward consumption.

**Figure 4.**
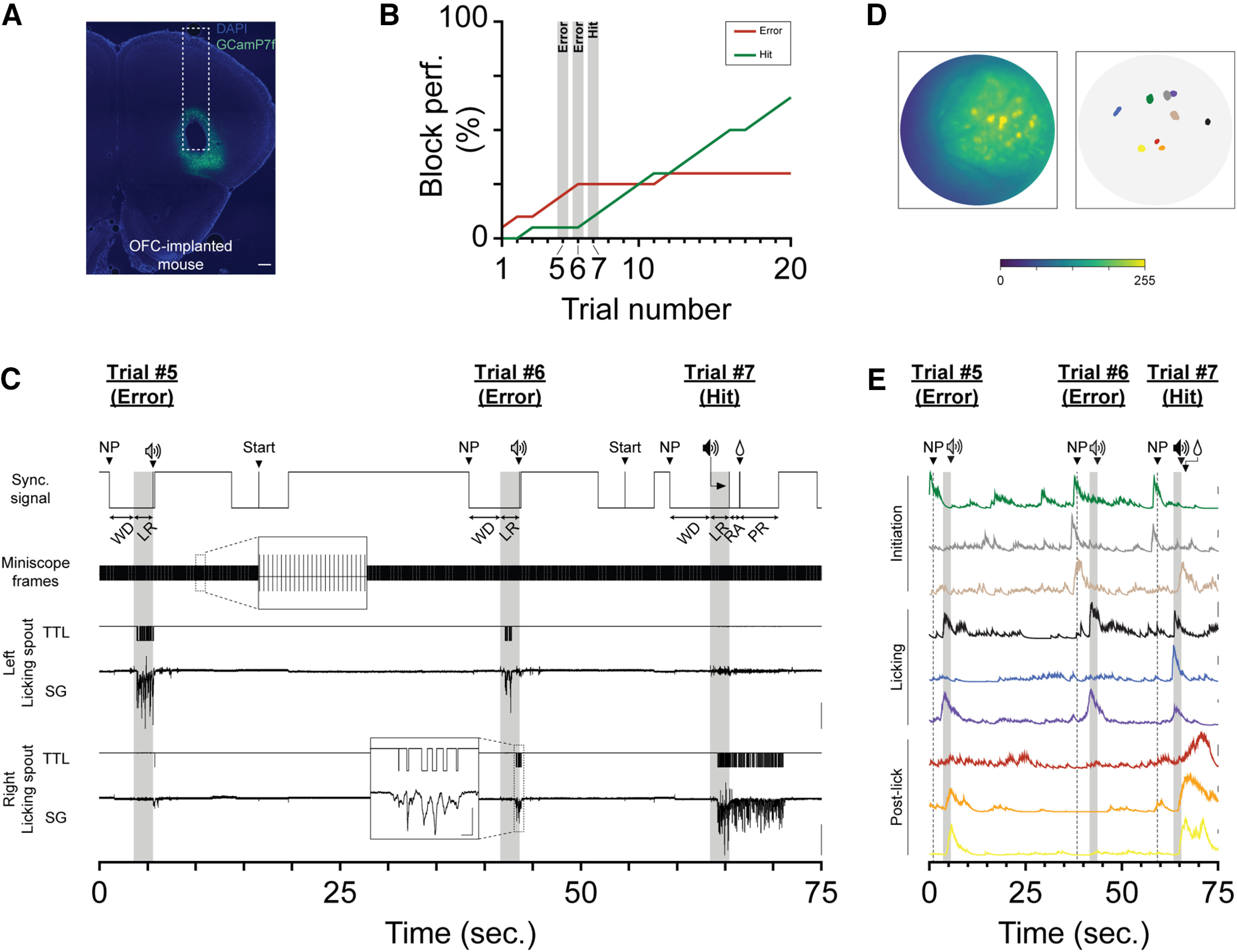
Combining 1-photon imaging of OFC with FreiBox. ***A***, GCaMP7f expression and GRIN lens track in OFC. Scale bar, 250 μm. ***B***, Block performance of the implanted mouse in ***A*** during the discriminative learning. The 3 highlighted trials (5, 6, and 7) are the same as shown in ***C*** and ***E***, and the shaded areas represent the LR windows. ***C***, Simultaneous recordings of the Miniscope and FreiBox signals used to synchronize calcium activity with behavioral events. The shaded areas indicate the LR windows. Extended Data Figure 4-1 describes the active commutator used to perform Miniscope recording in a freely moving animal. ***D***, ***E***, Examples of 9 modulated neurons (***D***) and their calcium traces (***E***) from the video recorded in ***C*** during the same trial sequence as shown in ***B***. ***D***, Left, Maximum projection picture obtained on the first 10,000 frames of the recording session. ***D***, ***E***, The spatial footprints (***D***, right) and the calcium traces (***E***) were obtained with the open-source pipeline CalmAn (gSig/gSiz: 3/13). The calcium traces represent the denoised temporal traces (CalmAn parameter C; scale bar, 10 dF) and were colored in accordance with their spatial footprints in ***D***. The shaded area in ***E*** indicates the LR windows. Extended Data Figure 4-2 shows similar calcium activity patterns recorded in OFC of a Thy1-GCaMP6f mouse during serial RL.

10.1523/ENEURO.0469-22.2023.f3-1 Figure 3-1 Individual behavioral data and serial reversal learning setting. ***A***, ***B***, Number (***A***) and average (***B***; mean ± SEM) of trials to reach the criterion of each individual mouse used in Figure 4. For mice 1 (*n* = 7, *t* = –2.492, *p *=* *0.047), 2 (*n* = 7, *t* = –3.739, *p *=* *0.01), 3 (*n* = 6, *t* = –6.100, *p *=* *0.002), 4 (*n* = 8, *t* = –4.633, *p *=* *0.002), and 5 (*n* = 8, *t* = –4.757, *p *=* *0.003), a paired *t* test was performed to compare the numbers of trials to criterion during DL and RL blocks. In contrast, Wilcoxon signed-rank tests were performed for the mice 6 (*n* = 7, *z* = 2.201, *p *=* *0.031), 7 (*n* = 7, *z* = 2.201, *p* = 0.031), and 8 (*n* = 7, *z* = 2.197, *p* = 0.031). * and ** show significance at the risk α = 0.05 and 0.01, respectively. ***C***, ***D***, By changing the number of blocks in the GUI (***C***), we are able to control a serial intrasession RL in well trained Thy1-GCamP6f mice as shown in ***D***. OFC GCamP6f expression as well as Ca^2+^ imaging recorded during the same behavioral session are shown in Extended Data Figure 4-2. Download Figure 3-1, TIF file.

### Behavioral training

To train each mouse to perform the task, we slowly introduced each task component and personalized the training to respect the individual learning rate. The first stage consisted of the association among the licking spouts, the water reward, and the auditory cues. To accomplish this, the LC gate was left opened until the mouse placed its head in the LC. If the mouse licked on the spout, the pure tone cue (5 kHz, 85 dB) was played immediately, the reward was delivered, and the gate closed after 4 s. In contrast, if the mouse did not lick on the spout 2 s after the HE (missed trial), the white noise cue (85 dB) was played and the gate was closed. At this stage, until stage 3, only one spout was presented in the LC and a session for each licking spout was programmed to avoid a spatial bias. The second stage was similar, except that the licking response and anticipatory response were introduced. In the third stage, the NP was introduced, meaning that the LC gate was kept closed until the mouse performed a NP. During the fourth stage, we introduced the DL by presenting both spouts in the LC. To help the animal, we used immediate feedback by playing the white noise cue immediately after the mouse licked on the wrong spout, followed by LC gate closing. In the next training stage, the final paradigm was used to perform intersession reversal of DL session. In such configuration, the reward spout learned during DL in the morning is reversed for the afternoon sessions. Finally, the intrasession reversal task was used. At this final stage, only one session per day was used to maintain a high motivation level in the animals. With this protocol, the mice learned the task in a few weeks. For instance, the training of the cohort used for data provided in Figure 3 lasted on average 30 ± 2 sessions (*n* = 8 mice), which corresponds to ∼3 weeks when performing two sessions per day on 5 training days per week.

### Surgery

All animal procedures were performed in accordance with the guideline RL 2010/63/EU and approved by the Regierungspräsidium Freiburg. General anesthesia was administered using a mixture of oxygen and isoflurane (induction, 3–5%; maintenance, 1.5–3%; CP Pharma), associated with a subcutaneous buprenorphine injection (0.05 mg/kg; Temgesic). The mice were then fixed on a stereotaxic frame (model 942, Kopf) and placed on heating blanket (Rodent Warmer X1, Stoelting). During surgery, the depth of anesthesia was determined by the extinction of the pain reflexes (intertoe and eyelid closure reflexes), and the eyes were protected from dehydration with eye ointment (Bepanthen; catalog #798–037, Henry Schein Medical). Five minutes before the rostrocaudal skin incision, a local surface lidocaine anesthesia (xylocaine gel, 2%; catalog #1138060, Shop-Apotheke) was applied to the scalp. To maintain the fluid balance, supplementation with 1 ml of Ringer’s solution was performed every 2 h.

For 1-photon calcium imaging in C57BL/6J mice, a virus injection procedure (De La Crompe et al., 2020) was performed right before gradient-index (GRIN) lens implantation. After craniotomy, a virus solution (rAAV1-hSyn-jGCamP7f; catalog #104488-AAV1, Addgene) was injected under stereotaxic conditions (400 nl, 3 × 10^12^ viral genomes/ml) into OFC [anteroposterior (AP), +2.5 mm from bregma; mediolateral (ML), +1.5 mm from midline; dorsoventral (DV), −1.8 mm from cortical surface] using glass microcapillary (tip diameter, approximately 35 μm; 1–5 μl Hirschmann microcapillary pipette; catalog #Z611239, Sigma-Aldrich) connected to a custom Openspritzer pressure system (Forman et al., 2017). To perform GRIN lens implantation, we followed the instructions provided by the Miniscope V4 group (http://miniscope.org/index.php/Online_Workshop). After craniotomy and a tunneling procedure using 25 gauge needle, the GRIN lens (diameter, 0.5 mm; pitch, 2; catalog #1050–002183, Inscopix), preassembled to the baseplate, was inserted above the OFC (AP, +2.3 mm from bregma; ML, +1.5 mm from midline; DV, −1.8 mm from cortical surface). The lens was then secured by using a light-blocking cement obtained by mixing a small amount of a black pigment (black iron oxide; catalog #832036, Kunstpark) with a dental cement (Paladur, Kulzer). To build the mounted GRIN lens and adjust the working distance, we used a custom Miniscope holder (https://github.com/Optophys-Lab/FreiBox) in combination with a lens holder available on the github site for Moorman’s laboratory (https://github.com/moormanlab/miniscope-goodies) before cementing the GRIN lens to the baseplate. At the end of surgery, a cap was secured to the baseplate to protect the lens. All Miniscope materials were purchased at the Open Ephys store or were 3D printed with a Ultimaker 3D printer and black PLA filament.

To prevent postoperative pain, we performed subcutaneous injections of buprenorphine (0.05 mg/kg every 6 h; Temgesic) and carprofen (5 mg/kg every 24 h; Rimadyl, Zoetis) during the next 3 d postsurgery. To continue the analgesic treatment during the night, we provided a buprenorphine (Temgesic) solution (0.1 g/L) mixed in drinking water containing 5% (w/v) d-glucose (100 ml; catalog #G8769, Sigma-Aldrich) to mask the drug taste. The weight and the general condition of the animals were monitored daily in the 4 d following surgery and weekly after the recovery of the animal.

### Miniscope recording and data analysis

Before starting the Miniscope recordings, trained animals were habituated to hold a dummy 3D-printed Miniscope during two to three sessions (https://github.com/Aharoni-Lab/Miniscope-V4). Once habituated, the mice were placed in the behavioral box with the Miniscope secured to the implanted baseplate. During the behavioral sessions, Open Ephys recordings (sampling rate, 30 kHz) were performed simultaneously with calcium imaging to synchronize, with the same clock system, the data streams coming from the FreiBox (Fig. 4*C*, synchronization signal) and the Miniscope V4 (Fig. 4*C*, Miniscope frames). After the acquisition (LIFEBOOK U749 laptop, Fujitsu), the videos were preprocessed to remove the noise produced by the electrowetting lens driver (https://github.com/Aharoni-Lab/Miniscope-V4/wiki/Removing-Horizontal-Noise-from-Recordings) and cropped to preserve the part of the field of view containing only the GRIN lens. We then used the open-source tool library CaImAn (gSig/gSiz: 3/13; Giovannucci et al., 2019) to extract the spatial footprints and their associated temporal calcium traces, expressed as denoised temporal traces (parameter C of the source extraction algorithm CNMF-E). All the video-processing steps were made with Python, and the following analyses were performed in MATLAB. To find behavioral correlations of the calcium traces, we first aligned all behavioral events to the closest recorded frames time stamp. We then calculated the waveform average of the median absolute deviation (MAD) of the denoised temporal traces. Based on this approach, we defined units as “modulated” when their averaged calcium peaks crossed a 3× MAD threshold. Finally, we classified modulated footprints as initiation, licking, or postlick neurons if their averaged MAD peaks occurred during the WD, the licking response, or RA period, respectively.

10.1523/ENEURO.0469-22.2023.f4-1 Figure 4-1 The FreiBox commutator. ***A–C***, Neurophysiological recordings in freely moving animals require untangling of the Miniscope coaxial cables connected to the freely moving implanted animal. To solve that problem, previous designs developed a well established method to detect the rotation of a cable implant by tracking the relative position of a magnet to a sensor called a Hall sensor (Fee and Leonardo, 2001; Liberti et al., 2017; Barbera et al., 2020). By modifying a commutator from the Gardner laboratory (Liberti et al., 2017), we designed a low-cost (111 €) open-source commutator as described in the exploded view (***A***). By adding a magnetic ring rotary encoder (***B***), we improved the detection of the cable position and constrained the cable movement. This design offers the advantage of maintaining the magnet close to the detector ring even when the connected mouse moves in a spacious environment. The schematic representation of the ring commutator (***C***) shows that a magnet glued to the Miniscope coaxial cable can rotate along the cable axis among the 3 fixed Hall sensors arranged in a circle around the cable (H1, H2, and H3). ***D***, To track the position of the magnet into the ring detector, the direction field (DF) of each Hall sensor is calculated as described by an Arduino using the function analogRead (“aR”). ***E***, ***F***, By combining the direction fields of the 3 Hall sensors (***E***), it is possible to define the orientation of the magnet in the field forms by the 3 Hall sensors (among 6 radial positions) and adjust the cable position on the basis of a decision matrix (***F***). ***G–I***, To build the FreiBox commutator, the 3 Hall sensors (***G***) and a motor driver card (***H***) are connected to the step motor and the Arduino; the signals from the Miniscope and the Hall sensors are relayed via a slip ring (***I***). ***J***, To test whether the commutator rotation induces additional data loss during Miniscope recordings, we compared the percentage of frame loss when the Miniscope was connected to the DAC board with a simple coaxial cable and left immobile on a table (“Cable only”; *n* = 12 sessions), to acquisitions performed during behavioral sessions (18 sessions, *n* = 3 mice). More precisely, we calculated the frame loss percentage when the commutator was actively rotating (“Beh. Rotation”) or not (“Beh. Only”) during imaging sessions in mice performing the task. As illustrated in the box-and-whisker plots (***J***), we found that the basal percentage of frame loss (Cable only) did not statistically increase when the signal is transmitted via an inactive commutator (Behavioral Only vs Cable only, 0.806 ± 0.444 vs 0.715 ± 0.238; *n* = 18 vs 12; Mann–Whitney rank-sum test: *U* = 88.00, *p *=* *0.406), or during active rotation (Behavioral rotation vs Cable only, *n* = 18 vs 12; Mann–Whitney rank-sum test: *U* = 66.500, *p *=* *0,060), or when active rotation is compared with inactive condition (Behavioral Rotation vs Behavioral only, 0.371 ± 0.158 vs 0.715 ± 0.238; *n* = 18; Wilcoxon signed-rank test: *z* = –0.973, *p *=* *0.358). Download Figure 4-1, TIF file.

10.1523/ENEURO.0469-22.2023.f4-2 Figure 4-2 OFC calcium imaging during intrasession RL. ***A***, Thy1-GCaMP6f expression and GRIN lens track in OFC. Scale bar, 250 μm. ***B***, ***C***, Examples of three modulated neurons (***B***) and their calcium traces displayed as column (***C***) aligned to the head entry in the LC (0 s) and split between hit, error, and miss trials during the same behavioral session as shown in Extended Data Figure 3-1*D*. The maximum projection picture (***B***, left) was obtained on the first 10,000 frames of the recording session. The spatial footprints (***B***, right) and the normalized calcium traces (***C***) were obtained with the open-source pipeline CalmAn (gSig/gSiz: 3/13). The calcium traces represent the normalized denoised temporal traces (CalmAn parameter C; scales, 5 normalized df) and were colored in accordance with their spatial footprints shown in ***B***, right. Normalized calcium traces were aligned to the beginning of the LR windows (shaded in gray), sorted by trial type (hit, error, or miss) and their trial number (shown here in an ascending order). The colored dots indicate the nose poke time stamps. For the hit trials, the dashed lines indicate the end of the anticipatory response windows, immediately followed by a water reward delivery. Download Figure 4-2, TIF file.

### Transcardiac perfusion and histologic control

After completion of the experiments, the mice received an overdose of a pentobarbital sodium (1 ml; 1.6 g/L, i.p.; Narcoren). After respiratory arrest, a transcardiac perfusion (PBS 0.01 mm followed by PBS 0.01 mm/formaldehyde 4%) was performed for histologic validations. The brain was kept overnight in a solution of PBS 0.01 mm (catalog #70011–051, Thermo Fisher Scientific)/formaldehyde 4% (v/v; catalog #E15711, Science Services), cryoprotected several days in a solution of PBS 0.01 mm/sucrose 30% (w/v; catalog #1076511000, Merck Millipore), and then cut into 50 μm slices with a sliding microtome (catalog #SM2010 R, Leica Biosystems). The images were acquired with an Axioplan 2 microscope (Zeiss) controlled by the software Axiovision (version 4.8, Zeiss). Shading correction and stitching were performed with Fiji (based on ImageJ from National Institutes of Health) using the BaSiC (Peng et al., 2017) and Grid/Collection plugins, respectively.

### Figure design and statistical analysis

All plots were generated with MATLAB and Python and were assembled with Illustrator CS6 (Adobe) to generate the figures. For Figure 3*A* and Extended Data Figure 3-1*D*, the mouse head drawing was downloaded from https://scidraw.io/. The statistical analyses were performed (De La Crompe et al., 2020) using SigmaPlot 12 (Systat Software). For independent samples, we applied the normality (Shapiro–Wilk test) and equal variance tests. A *t* test was used to compare samples if they were normally distributed and their group variances equal. Otherwise, the Mann–Whitney signed-rank test was performed. For dependent samples, the paired *t* test was used for normally distributed paired samples. In contrast, when the normality distribution test failed (Shapiro–Wilk test, *p *<* *0.05), the Wilcoxon signed-rank test was performed. In same way, Friedman repeated-measures ANOVA on ranks was used instead of one-way repeated-measures ANOVA, when normality distribution was not verified. ANOVA was then followed by Tukey’s *post hoc* test.

## Results

### Validation of the strain gauge lickometer

Using licking as a behavioral readout requires measurement of the force applied on the spout with a high sensitivity. Although it has been shown that SG lickometers are able to detect licks when a mouse is drinking on a cup (Wang and Fowler, 1999), it has never been tested in a configuration where the SG is connected to a spout. Hence, before integrating our competitive SG lickometer, we validated its sensitivity by comparing the SG-offline and SG-online methods with the well established TS (Fig. 2*D*; one-way repeated-measures ANOVA: *n* = 6, *F* = 4.906, *p* = 0.033). In doing so, we found that the sensitivity of the SG-offline method is comparable to TS (TS vs SG-offline, 0.685 ± 0.0676 vs 0.740 ± 0.0374, Tukey’s test; TS vs SG-online, *q* = 33.073, *p* = 0.125) and significantly higher than SG-online (SG-online vs SG-offline: 0.546 ± 0.0372 vs 0.740 ± 0.0374, *n* = 6, Tukey’s test; TS vs SG-online, *q* = 34.30, *p* = 0.031). In contrast, the TS and SG-online shared a similar sensitivity level (TS vs SG-offline: *n* = 6, Tukey’s test, *q* = 31.227, *p* = 0.672). The PPV was not significatively different between the methods (Extended Data Fig. 2-1*D*; TS vs SG-offline vs SG-online; one-way repeated-measures ANOVA: *n* = 6, *F* = 0.166, *p *=* *0.850).

We tested whether the sensitivity difference between the offline and online SG methods can be compensated for by lowering the value of the detection threshold. As illustrated in Figure 2*B* and Extended Data Figure 2-1*A*, the licks with a smaller amplitude did not cross the voltage threshold. By artificially adjusting the detection levels (Extended Data Fig. 2-1*A*,*B*), we showed that a reduction of only 20 mV is sufficient to improve the sensitivity greatly without compromising the PPV (Extended Data Fig. 2-1*C*,*D*). The potential to fine-tune the detection threshold greatly improves the real-time detection performance. We implemented an Arduino oscilloscope (Extended Data Fig. 2-1*E*, modified from https://create.arduino.cc/projecthub/aimukhin/advanced-oscilloscope-955395) to monitor the SG-signals and their thresholds online. Together, these data demonstrate that the SG lickometer can be successfully implemented to read out online licking behavior.

### Behavioral training and serial RL

After having validated the ability of the SG lickometer to track individual licks, we programmed FreiBox to control an intrasession RL task (Fig. 3*A*,*B*). The results were obtained with a cohort of eight mice trained during 4–6 weeks to acquire the intrasession reversal. As illustrated in their individual (Fig. 3*C*, Extended Data Fig. 3-1*A*,*B*) and best (Fig. 3*D*) sessions, the mice were able to learn and reverse very quickly. Indeed, by using a learning criterion of 70% correct licking in a sliding average window of five trials, the mice succeeded in both DL and RL blocks in <10 and 20 trials, respectively. On average (Extended Data Fig. 3-1*B*), the mice required more trials to succeed in RL than in DL, a feature that has been described earlier (Castañé et al., 2010). Based on those positive results, we refined the FreiBox capability to control multiple task variants via an easy-to-use Arduino–Python graphical user interface (GUI) interface (Extended Data Fig. 3-1*C*). With such improvements, we were able to perform serial RL (Extended Data Fig. 3-1*D*) or probabilistic RL (Parker et al., 2016) by simply adjusting the block number or reward probability, respectively.

### Calcium imaging during discriminative learning

DL paradigms have often been used under 2-photon conditions to explore the role of cortical areas in decision-making and motor execution (Chen et al., 2017; Guo et al., 2017; Galiñanes et al., 2018; Morrissette et al., 2019; Wu et al., 2020). To test the feasibility of coupling calcium imaging in FreiBox with such a behavioral task, we developed an active Miniscope commutator (Extended Data Fig. 4-1) and trained a mouse injected with the viral vector rAAV1-hSyn-jGCamP7f and implanted with a GRIN lens in OFC (Fig. 4*A*). Once the DL was complete, we performed recordings with the Miniscope (Fig. 4*B*,*C*). To synchronize calcium data with behavior, we recorded simultaneously with an Open Ephys system, the analog and TTL SG licking signals, the Miniscope frames TTL from the digital-to-analog converter (DAC) box as well as the synchronization signal from the FreiBox (Fig. 4*C*). Figure 4*C* shows an example of those signals recorded from two consecutive error trials (Errors #1 and #2) followed by a hit attempt (Hit). That trial sequence was the turning point of the session because afterward the hit performance continuously increased while the error rate stagnated (Fig. 4*B*).

To analyze calcium videos, we used the open-source tool library CaImAn (Giovannucci et al., 2019) to extract the spatial footprints (Fig. 4*D*) and their associated temporal calcium traces (Fig. 4*E*). After finding some calcium activities modulated during recorded behavior (see Materials and Methods), we aligned neuronal examples to behavioral events (Fig. 4*E*) and found that most of the modulated jGCamP7f-OFC-expressing neurons can be separated into three main populations, depending on their peak activity in the task periods, as follows: initiation (during WD), licking (during licking response), or postlick (during an RA period). We confirmed this response distribution by repeating the same experiment in two trained Thy1-GCaMP6f mice during serial RL, as illustrated for one of them in Extended Data Figures 3-1*D* and 4-2. These results illustrate the complex encoding of OFC neurons during goal-directed behavior and the ability of our task to dissect different neuronal processes.

## Discussion

Since the emergence of head-restraint preparations, directional licking has been used as a behavioral readout to access numerous cognitive processes including short-term memory (Wang et al., 2021), decision-making (Wu et al., 2020), or motor preparation (Chen et al., 2017). Conventional approaches for monitoring online licking behavior use electrical and optical sensors (Weijnen, 1998; Williams et al., 2018) or high-speed video recordings combined with deep-learning approaches (Bollu et al., 2021; Catanese and Jaeger, 2021). While those lickometers are very efficient, the behavioral measurements are limited to the occurrence of the licks and cannot provide information about the force or vigor of these movements (Dudman and Krakauer, 2016). To overcome those limitations, we developed and validated a cost-effective and sensitive force lick sensor, which can be used with freely moving mice. In contrast to the available force lickometer which measures licking on a flat disk (Rudisch et al., 2022; Wang and Fowler, 1999), our SG lickometer can be easily integrated to measure directional licking in a large number of behavioral setups, including head-fixed settings. The SG lickometer gives the opportunity to correlate an unexplored aspect of licking behavior (i.e., the licking force) in both head-fixed and freely moving conditions, thereby allowing evaluation of movement vigor (Dudman and Krakauer, 2016) and measurement of the motivation of the animal to collect rewards (Deng et al., 2021).

Based on our novel FreiBox platform, we are able to combine freely moving licking-based behaviors (e.g., DL, RL, or set shifting) with the most recent optogenetic approaches (Table 1). As a proof of concept, we performed Miniscope calcium imaging in mice performing DL and RL. We recorded several response patterns in the OFC, demonstrating that our platform is able to capture complex neuronal dynamics reflecting several aspects of behavioral flexibility such as decision-making, motor planning, and action evaluation. We recorded calcium activity related to nose poking and licking, but also to a neuronal population expressing a long-lasting plateau following reward delivery. These findings corroborate results observed with electrophysiological striatal and cortical recordings of monkeys performing an RL task (Pasupathy and Miller, 2005; Histed et al., 2009). Altogether, these data introduce FreiBox as a useful tool to explore the neuronal substrates of behavioral flexibility underlying directional licking in rodents.

Adaptation of the methods to experimental questions is a major advantage of developing open-source tools (White et al., 2019). Our development of FreiBox and its low-cost parts represent complementary additions to the recently emerging battery of open-source techniques (Hou and Glover, 2022; White et al., 2019). Recently, open-source initiatives such as Miniscope (Aharoni et al., 2019) and Open Ephys (Siegle et al., 2017) have opened the gate to the standardization of flexible, low-cost, and open-source technologies. In a global context of budget reduction, open-source projects and publications constitute a great advance to reduce the financial dependency of the laboratories without compromising research quality.

**Table 1:** Price list for assembling the FreiBox platform

ID	Name	Price (€)
1	FreiBox	903
2	Commutator	111
3	Tools	160
4	Miniscope UCLA V4	1580
5	Open Ephys system	3479
6	Laser controller	900
7	Computer	1500
	Total	8633

A precise list of the components and providers is available at https://github.com/Optophys-Lab/FreiBox. The prices were updated in June 2022.

## Synthesis

Reviewing Editor: Silvia Pagliardini, University of Alberta Decisions are customarily a result of the Reviewing Editor and the peer reviewers coming together and discussing their recommendations until a consensus is reached. When revisions are invited, a fact-based synthesis statement explaining their decision and outlining what is needed to prepare a revision will be listed below. The following reviewer(s) agreed to reveal their identity: Eric Yttri, Balazs Hangya.

The Authors present an open source behavioral setup for detecting mouse licks while training mice on a discrimination task, which they combine with 1-photon imaging. This open source, affordable setup might be useful for many labs. This methods manuscript is remarkably well-written and well-described. I applaud the author’s thoroughness at rigorously testing and demonstrating performance - both of the components and in real-world examples.

The presentation could be improved at several points to help the readers, that is, the potential users of this tool: my detailed comments are below.

If the paper does have a fault, it is that the product itself is not that novel. Rather, it is a conglomerate of mostly-well used tools. This isn’t a fatal flaw in my opinion. However, a few more citations might be of use in order to put the document in a larger context. eNeuro in particular has had some excellent papers including Mazziotti 2020, Belsey 2021, Jun Ho Lee 202, even Hou 2022 - come to mind both for their tasks and their focus on being thoroughly documented open-source tools and the importance of this for the field.

In particular, Vassilev 2022 would be a good paper to compare and contrast to, as it has many similarities. With the papers trying to obtain similar outcomes, this is a great opportunity to demonstrate why Freibox should be used.

1.The argument for noise-inducing lickometers is a rather weak strawman. This is especially at issue because the paper that focuses on o-phys rather than e-phys, so we don’t get a hands-on sense of things. Many groups use all manner of lick ports without a clear issue. Could the authors provide better documentation of this being an issue? I don’t find it critical to greatly extend, but it would make for a much stronger argument

2. ’With this protocol, the mice learned the task in a few weeks.’ Please quantify what you mean by ‘a few weeks’.

3. Are the design files for all 3D-printed parts made public?

4. The lick detection sensitivities reported in the first paragraph of results are rather low for all methods tested. Also, the reported values don’t seem to match those on Fig.2E - please double check (TS and SG-online swapped?). The Authors comment on improving this by lowering the detection threshold. While this is demonstrated in Extended Data Fig. 2-1, it would be worth elaborating on what sensitivity values can be reached with what corresponding PPV values in the main text as well. It would also be informative to include the inter-lick distribution for the ground truth licks in Fig.2D. What is the main reason for the false detections?

5. Please also show typical performance, not only best performance (best sessions averaged in Fig.2C) for RL performance.

6. Please improve the resolution of the figures. For instance, the labels in Extended Data Fig. 3-1 are impossible to read. In the legend, ‘Supplement S4’ is referred to; should that be Extended Data Fig. 4-3? I don’t see Extended Data Fig. 4-2. There is also a reference to ‘Supplement S3D’, probably from a previous version.

7. It is unclear how Extended Data Fig. 4-3C shows 3 examples - I only see separation by trial type and alignment. Also, please clarify why we see ‘licking’ in Miss trials, which is claimed to stand for ‘entry in the LC without licking on a spout’.

8. ‘Reward neurons’ sounds like a confusing term for neurons active in the ‘reward anticipation’ period. One might assume it refers to neurons responsive to reward delivery.

9. Please resolve FOV (I assume field of view).

10. Please clarify what you mean by ‘classic touch sensor’.

11. Please provide the sound intensity of the stimuli.

12. How many trials did the animals perform per session? Was this number predetermined? Can a higher number of trials be achieved?

13. Would it be possible to use more than two licking spouts (e.g. for sequence learning)? Is there a limitation from the Arduino side?

14. Typos: Introduction, second paragraph, fifth line ‘... in head restrained mice AND in a freely moving animal.’; Animals and water restriction protocol, first paragraph, last line ‘... explore the calcium activity of the orbitofrontal cortex (OFC) during DC’ - did you mean DL?

15. Statistics: I find it important to note that normality tests never ‘prove’ normality of a distribution, as the only statistical conclusion they can provide is a significant deviation from normality (a test being not significant is not conclusive). Therefore, the practice of deciding between parametric and non-parametric tests based on the outcome of normality tests is not well grounded, statistically speaking. However, I realize that this has become a common practice and I assume that a more consistent use of the tests wouldn’t dramatically change the results in this case; therefore, I don’t deem fixing this issue absolutely necessary for accepting the paper.

16. This sentence is difficult to parse: As illustrated in their best (Fig. 3C) and the individual (Extended Data Fig. 3-1A,B) sessions, the mice were able to reach 70% of correct licking in a sliding average window of 5 trials, in fewer than 10 and 20 trials during the DL and RL blocks respectively.

17. Figures. I’m not sure that fig1c helps, especially given its size. If e-h would benefit from being afforded more size (I do), I’d cut C

fig2a is almost so grainy as to be uninterpretable

fig 2e, asterisk is off center

fig 3, as well as throughout, many font sizes are used, and some are a bit blurry. for publication, please spend a bit of time on the aesthetics

Fig.4B rather unclear.

## Author Response

Synthesis Statement for Author (Required):

The Authors present an open source behavioral setup for detecting mouse licks while training mice on a discrimination task, which they combine with 1‐photon imaging. This open source, affordable setup might be useful for many labs. This methods manuscript is remarkably well‐written and well‐ described. I applaud the author’s thoroughness at rigorously testing and demonstrating performance ‐ both of the components and in real‐world examples.

The presentation could be improved at several points to help the readers, that is, the potential users of this tool: my detailed comments are below.

If the paper does have a fault, it is that the product itself is not that novel. Rather, it is a conglomerate of mostly‐well used tools. This isn’t a fatal flaw in my opinion. However, a few more citations might be of use in order to put the document in a larger context. eNeuro in particular has had some excellent papers including Mazziotti 2020, Belsey 2021, Jun Ho Lee 202, even Hou 2022 ‐ come to mind both for their tasks and their focus on being thoroughly documented open‐source tools and the importance of this for the field.

In particular, Vassilev 2022 would be a good paper to compare and contrast to, as it has many similarities. With the papers trying to obtain similar outcomes, this is a great opportunity to demonstrate why Freibox should be used.

We thank the reviewer for the additional literature suggestions. We have now added the references in the introduction and rephrased the second paragraph of the introduction as follow: “Typical behavioral responses in freely moving animals are nose pokes (NP) in specific chambers, visits in maze arms, presses on a specific lever, or movements of a joystick in a specific direction to obtain a reward (Belsey et al., 2020; Castañé et al., 2010; Izquierdo et al., 2017; Lee et al., 2020; Mazziotti et al., 2020; Parker et al., 2016; Vassilev et al., 2022).

For instance, Vassiliev et al. 2022 recently developed a well‐documented and open‐source setup to perform a Go/No Go‐task. While this approach is already very useful to explore behavioral flexibility in freely moving mice, no currently available setup permits measurement of directional licking, a behavioral paradigm which can access numerous cognitive processes in head‐restrained mice (Bollu et al., 2021; Catanese & Jaeger, 2021; Mayrhofer et al., 2019; Y. Wang et al., 2021; Wu et al., 2020a). Such a tool would provide the opportunity to perform translational studies between head‐fixed and freely‐moving conditions. Focusing on licking behavior, we have designed a fully open‐source and cost‐ efficient (∼900 €) behavioral platform to explore behavioral flexibility in freely moving mice. This new box, called FreiBox (Fig. 1), is capable of monitoring a wide variety of licking‐based tasks, such as discriminative learning (DL) and serial reversal learning (RL), by tracking online licks with strain‐gauge‐based force lickometers.”

We also added the ‘Hou et al. 2022’ reference in the last paragraph of the discussion as follow: “Our development of FreiBox and its low‐cost parts represent complementary additions to the recently emerging battery of open‐source techniques (Hou & Glover, 2022; White et al., 2019).”

1. The argument for noise‐inducing lickometers is a rather weak strawman. This is especially at issue because the paper that focuses on o‐phys rather than e‐phys, so we don’t get a hands‐on 2 sense of things. Many groups use all manner of lick ports without a clear issue. Could the authors provide better documentation of this being an issue? I don’t find it critical to greatly extend, but it would make for a much stronger argument

This is a very valid comment. Unfortunately, we have not yet performed electrophysiological recordings in FreiBox. However, since we believe that the strength of the SG‐lickometer is to provide another interesting behavioral information, the licking force, we have modified the first paragraph of the discussion as follow to address the reviewer comment: “Since the emergence of head‐restraint preparations, directional licking has been used as a behavioral readout to access numerous cognitive processes including short‐term memory (Y. Wang et al., 2021), decision‐making (Wu et al., 2020b), or motor preparation (Chen et al., 2017). Conventional approaches for monitoring online licking behavior use electrical and optical sensors (Weijnen, 1998; Williams et al., 2018) or high‐speed video recordings combined with deep‐learning approaches (Bollu et al., 2021; Catanese & Jaeger, 2021).

While those lickometers are very efficient, the behavioral measurement is limited to the occurrence of the licks. To overcome those limitations, we developed and validated a cost‐ effective and sensitive force lick sensor which can be used with freely moving mice. In contrast to the available force lickometer which measures licking on a flat disk (Rudisch et al., 2022; Wang & Fowler, 1999), our SG‐lickometer can be easily integrated to measure directional licking in a large number of behavioral setups, including head‐fixed settings. In the future, the SG‐lickometer will give the opportunity to correlate an unexplored aspect of the licking behavior, the licking force, in both head‐fixed and freely‐moving conditions.”

2. ‘With this protocol, the mice learned the task in a few weeks.’ Please quantify what you mean by ‘a few weeks’.

The mice of the cohort on the Fig. 3 needed on average 30 {plus minus} 2 sessions (n=8). With 2 sessions per days and 5 days per week, the training time was 3 weeks on average. We added the following sentence in the section ‘Behavioral training’: For instance, the training of the cohort used for data provided in Fig. 3 lasted on average 30 {plus minus} 2 sessions (n=8 mice) which corresponds to approximatively 3 weeks when performing 2 sessions per day on 5 training days per week.

3. Are the design files for all 3D‐printed parts made public?

Yes, you can access them on the anonymized github made for the double‐blinded review process (https://github.com/FreiBox/FreiBoxPaper). Later on, we will transfer them to our lab github. 3

4. The lick detection sensitivities reported in the first paragraph of results are rather low for all methods tested. Also, the reported values don’t seem to match those on Fig.2E ‐ please double check (TS and SG‐online swapped?).

The Authors comment on improving this by lowering the detection threshold. While this is demonstrated in Extended Data Fig. 2‐1, it would be worth elaborating on what sensitivity values can be reached with what corresponding PPV values in the main text as well.

It would also be informative to include the inter‐lick distribution for the ground truth licks in Fig.2D.

What is the main reason for the false detections?

The mean values between TS and SG‐online have been accidentally swapped in the manuscript. We apologize for this glitch. We fixed the problem by changing the values in the result section ‘Validation of the strain gauge lickometer’.

To verify that the sensitivity values were correctly measured in the first place, we repeated the quantification of the video data and confirmed the sensitivity values (mean {plus minus} SEM) for the 3 detection methods described (TS: 0.675 {plus minus} 0.0856; SG‐Offline: 0.723 {plus minus} 0.0403; SG‐ Online: 0.518 {plus minus} 0.0427). We believe that such ‘rather low’ value can be explained by the setup configuration itself, especially as both TS and SG‐lickometer measurements provide the same sensitivity order. Even without knowing the exact source of the low values, we think that the ‘proof‐of‐concept’ experiment described in the Fig.2 is still informative and helps us to prove the feasibility of recording directional licking behavior with our SG‐ lickometer. We included the inter‐lick distribution of the ground truth licks in figure 2D and modified the legend accordingly.

For the TS, false detection can be explained by an electrical artefact or when the mouse is in close contact or very close to the spout or water droplet. For the SG, false detection occurs only when the SG signal crosses the threshold, thus mainly when the mouse exerts a force pressure on the spout. We added a comment in the method section ‘Sensitivity evaluation of the strain gauge lickometer’ to clarify this point: “The PPV was quantified to compare the false detection rate occurring with both lickometers. For the TS, FP licks occurred following an electrical artefact or when the mouse was in close contact or in an immediate proximity to the spout or water droplet. For the SG, false detection occurred only when the SG signal crosses the threshold, thus mainly when the mouse exerts a force pressure on the spout.”

5. Please also show typical performance, not only best performance (best sessions averaged inFig.2C) for RL performance. Since the block length is dependent of the performance of the mice during each block of each session, it is impractical to average the performances of multiple mice, except in their ‘expert session’. The variance of the block lengths is the reason why we plotted the best session in Figure 3 and all individual performances for each mouse and block in the ‘Extended Data Figure 3‐1A and 3‐ 1B’. 4

To illustrate the block length variability, we added in Fig.3C three plots obtained from the same animal. To cover the mouse performance range and typical performances, we selected an intermediate session performance between the worst and an expert session.

In line with these changes, we modified the legend of Fig. 3 as follows: “(C). Typical session performance obtained from the same animal during three different sessions. An intermediate performance plot (blue, intermediate) has been selected to cover the performance range of the animal between its worst (red, beginner‐like) and best (green, expert) sessions. “

Additionally, we changed the referencing of the figure in the main text accordingly: ‐ In the ‘Behavioral task’ section, first line: (Fig. 3A‐D) ‐ In the Behavioral training and serial RL: But see also question #16

6. Please improve the resolution of the figures. For instance, the labels in Extended Data Fig. 3‐1 are impossible to read. In the legend, ‘Supplement S4’ is referred to; should that be Extended Data Fig. 4‐3? I don’t see Extended Data Fig. 4‐2. There is also a reference to ‘Supplement S3D’, probably from a previous version.

We apologize for the very low resolution of the figures. We now increased the resolution to 300 dpi.

We thank the reviewers for pointing out the incorrect figure referencing. We exchanged the Extended Data Fig. 4‐3 by Extended Data Fig. 4‐2 and corrected the referencing accordingly.

7. It is unclear how Extended Data Fig. 4‐3C shows 3 examples ‐ I only see separation by trial type and alignment. Also, please clarify why we see ‘licking’ in Miss trials, which is claimed to stand for ‘entry in the LC without licking on a spout’.

In the Extended Data Fig. 4‐3C, we presented the calcium activities of three different neurons, displayed as columns, recorded in the OFC of a transgenic Thy1‐GCaMP6f mouse (Extended Data Figure 4‐2A). Their spatial footprints are shown in B. The calcium traces are aligned to the head entry and sorted by trial types (hit, error and miss). For each neuron, or column, the rows represent a single trial. To improve the clarity of the legend, we modified the legend of the Extended Data Figure 4‐3 as follows: (B‐C). Examples of three modulated neurons (B) and their calcium traces displayed as column (C) aligned to the head entry in the LC (0 s) and split between hit, error and miss trials during the same behavioral session as shown in Extended Data Figure 3‐1D.

We also thank the reviewer for the very important comment on miss trials. Concerning the ‘licking’ in miss trials, the data presented in the ‘Extended Data Fig. 4‐3C’ are calcium traces and not licking signals. We first think that the expression of a calcium transient does not necessarily mean that the mouse actually licked. Indeed, our ‘hardware definition’ of miss is a trial for which mice did not lick during the 2s‐response windows (see comment below *). More precisely, after the LR windows, the 5 licking chamber gate is automatically closed, blocking the access to the spout. Thus, the activity recorded during the LR in miss trials does not seem to reflect the execution of licks but rather the motor planning: During hit and error trials, the calcium transients often start right before the head‐ entry of the animal in the licking chamber (Figure 4E and Extended Data Fig. 4‐3C), suggesting that such a neuronal population encode for a motor plan rather than a licking execution program. * To improve the clarity of the definition of miss trials, we added in the ‘Behavioral task section’: ‘(entry in the LC without licking on a spout during the 2s LR)’.

8. ‘Reward neurons’ sounds like a confusing term for neurons active in the ‘reward anticipation’ period. One might assume it refers to neurons responsive to reward delivery. According to the reviewer’s comment, we changed the term ‘reward’ into ‘post‐lick’ in the sections ‘Miniscope recording and data analysis’ and ‘Calcium imaging during discriminative learning’, as well as, in the ‘figures 3E’ and ‘Extended Data Fig. 4‐3B,C’.

9. Please resolve FOV (I assume field of view). Thanks for the remark, we corrected the text by adding ‘field of view’ and removed the abbreviation FOV as it is not used afterwards in the text or legend.

10. Please clarify what you mean by ‘classic touch sensor’. To clarify the term ‘classic touch sensor’, we added the following text in the method section ‘Sensitivity evaluation of the strain gauge lickometer’: “The term touch sensor (TS) refers to all methods and electronic circuits derived from the ‘Electronic Drinkometer’ published by Hill and Stellar in 1951. This device had been designed to detect a voltage or capacitive change when a rodent tongue touches the liquid; in other words, the animal becomes part of an electronic circuit (Schoenbaum et al., 2001) and ‘completes’ it during licking (Hill & Stellar, 1951). To test whether the sensitivity of the SG‐lickometer (Fig. 2) is comparable to such classic TS, we placed 6 water‐restricted mice in a water‐delivery chamber (20 cm x 20 cm x 20 cm), containing a single licking spout attached to a SG (Fig. 2A) and connected to the TS circuit (Goltstein et al., 2018).”

11. Please provide the sound intensity of the stimuli. The sound intensity was settled to 85dB for both the white noise and the pure tone cue. We added this information in the section ‘Behavioral training’. 12. How many trials did the animals perform per session? Was this number predetermined? Can a higher number of trials be achieved? At the beginning of each session, we predetermine a minimum number of trials per block (= blocks length) and a minimum number of blocks. For figure 3D, the block length was set to 15 trials and the block number to 2, referring to 30 trials per session for an optimal performance in expert mice. 6 However, those values can vary on the basis of the animal performance measured online. Indeed, if the mouse was not performing well, it remained in the current block and exceeded 30 trials per sessions, as illustrated in the individual performances (Extended Data Figure 3‐1A). This approach avoids introducing a reversal in mice which have not yet properly learned the contingencies during a block. Therefore, the reviewer is correct in assuming that the total number of trials can be higher than the predetermined minimal number.

To explain this point, we added in the method section ‘Behavioral task’ the following sentences: “After the learning phase, the reward spout is automatically changed and the mice need to adapt their behavior (Fig. 3A, RL phase). To prevent to reverse the contingencies while they were not properly learned, the RL phase occurred only if the mouse reached (1) a minimum number of 15 trials (block length), and (2) an online performance criterion (70% hit in a sliding average window of 15 trials).”

13. Would it be possible to use more than two licking spouts (e.g. for sequence learning)? Is there a limitation from the Arduino side? Definitively yes. It is possible to add more spouts since the Arduino mega has 54 Digital Input/Output pins and so far we are using only 21. The main limitation for adding more spouts will be the reading speed which will be reduced by several tens of microseconds per spout according to our results presented in the Extended Data Figure 1‐1. To respond to this question, we added in the method section ‘FreiBox platform and its operant instruments’ the following: “For instance, since the Arduino Mega board has half of it digital pins available in FreiBox, it is possible to include several other spouts to perform sequence learning, without comprising the reading speed of more than several tens microseconds per additional spout.”

14. Typos: Introduction, second paragraph, fifth line ‘... in head restrained mice AND in a freely moving animal.’; Animals and water restriction protocol, first paragraph, last line ‘... explore the calcium activity of the orbitofrontal cortex (OFC) during DC’ ‐ did you mean DL? Sorry for the typos. We corrected them in the text. 15. Statistics: I find it important to note that normality tests never ‘prove’ normality of a distribution, as the only statistical conclusion they can provide is a significant deviation from normality (a test being not significant is not conclusive). Therefore, the practice of deciding between parametric and non‐parametric tests based on the outcome of normality tests is not well grounded, statistically speaking. However, I realize that this has become a common practice and I assume that a more consistent use of the tests wouldn’t dramatically change the results in this case; therefore, I don’t deem fixing this issue absolutely necessary for accepting the paper. To check the consistency of the results, we analyzed the normally distributed data set of this study by using non‐parametric tests. We analyzed the data from three figures as described in the tables 7 below (Figure 2E, Extended Data Figure 2‐1D and Extended Data Figure 3‐1B). Thereby, we found that most of the non‐parametric tests on normally distributed data, reproduced the conclusion of the parametric tests, arguing that changing the tests would not dramatically change the result in our study (as the reviewer already assumed). Figure 2E and Extended Data Figure 2‐1D (PPV): Figure ANOVA one‐way repeated measure

(parametric, original analysis) Friedman repeated measures ANOVA on ranks (non‐parametric) Outcome Figure 2E, Sensitivity (n=6) F=4.906, p=0.033 X2 =6.333, p = 0.052 Different Extended Data Figure 2‐1D, PPV (n=6) F=5.523, p<0.001 X2 = 24.142, p = 0.001 Similar Extended Data Figure 3‐1B: Mouse ID Paired T‐test (parametric, original analysis) Wilcoxon Signed Rank Test (non‐parametric) Outcome

1 (n=7 sessions) t=‐2.492, p=0.047 z= 2.371, p=0.016 Similar

2 (n=7 sessions) t= ‐3.739, p=0.01 Z= 2.371, p=0.016 Similar

3 (n=6 sessions) t = ‐6.100, p=0.002 z = 2.207, p=0.031 Similar

4 (n=8 sessions) t= ‐4.633, p=0.002 z= 2.524, p=0.008 Similar

5 (n=8 sessions) t= ‐4.757, p=0.003 z= 2.207, p=0.031 Similar

16. This sentence is difficult to parse: As illustrated in their best (Fig. 3C) and the individual (Extended Data Fig. 3‐1A,B) sessions, the mice were able to reach 70% of correct licking in a sliding average window of 5 trials, in fewer than 10 and 20 trials during the DL and RL blocks respectively.

We rephrased the sentence and split it as follows:

As illustrated in their individual (Fig. 3C and Extended Data Fig. 3‐1A,B) and best (Fig. 3D) sessions, the mice were able to learn and reverse very quickly. Indeed, by using a learning criterion of 70% correct licking in a sliding average window of 5 trials, the mice succeeded in both DL and RL blocks in fewer than 10 and 20 trials, respectively.

17. Figures. I’m not sure that fig1c helps, especially given its size. If e‐h would benefit from being afforded more size (I do), I’d cut C We removed figure 1C, resized the figures 1E‐H, and re‐arrange the figures and legends. 8 fig2a is almost so grainy as to be uninterpretable This is fixed now. fig 2e, asterisk is off center The asterisk on figure 2e have been centered. fig 3, as well as throughout, many font sizes are used, and some are a bit blurry. for publication, please spend a bit of time on the aesthetics

We unified the font size and improve the readability.

Fig.4B rather unclear. We apologize, the previous notation (Error#1, Error#2 and Hit) were not clear. Instead, we now use their trial number (5,6 and 7) and removed the auditory cue symbol on top. We think those modifications make figure 4B clearer. Additionally, we added in the legend of Fig.4C the meaning of the shaded areas as follows: “(B). Block performance of the implanted mouse (A) during the discriminative learning. The 3 highlighted trials (5, 6 and 7) are the same as shown in C and E and the shaded areas represent the LR windows.”

To keep the rest of the panel in a uniform style, we also changed the names on top of figures 4C and

4D and added in the legend the meaning of the shaded area as follows: “(C). Simultaneous recordings of the Miniscope and FreiBox signals used to synchronize calcium activity with behavioral events. The shaded areas indicate the LR windows.” “(D‐E). Examples of 9 modulated neurons (D) and their calcium traces (E) [...] spatial footprints in (D). The shaded area in (E) indicates the LR windows. “
