# Supplementary figures and images for: FreiBox: A Versatile Open-Source Behavioral Setup for Investigating the Neuronal Correlates of Behavioral Flexibility via 1-Photon Imaging in Freely Moving Mice
Source: eNeuro. 2023 Apr 25;10(4):ENEURO.0469-22.2023. doi: 10.1523/ENEURO.0469-22.2023 (PMC10166259; doi:10.1523/ENEURO.0469-22.2023)

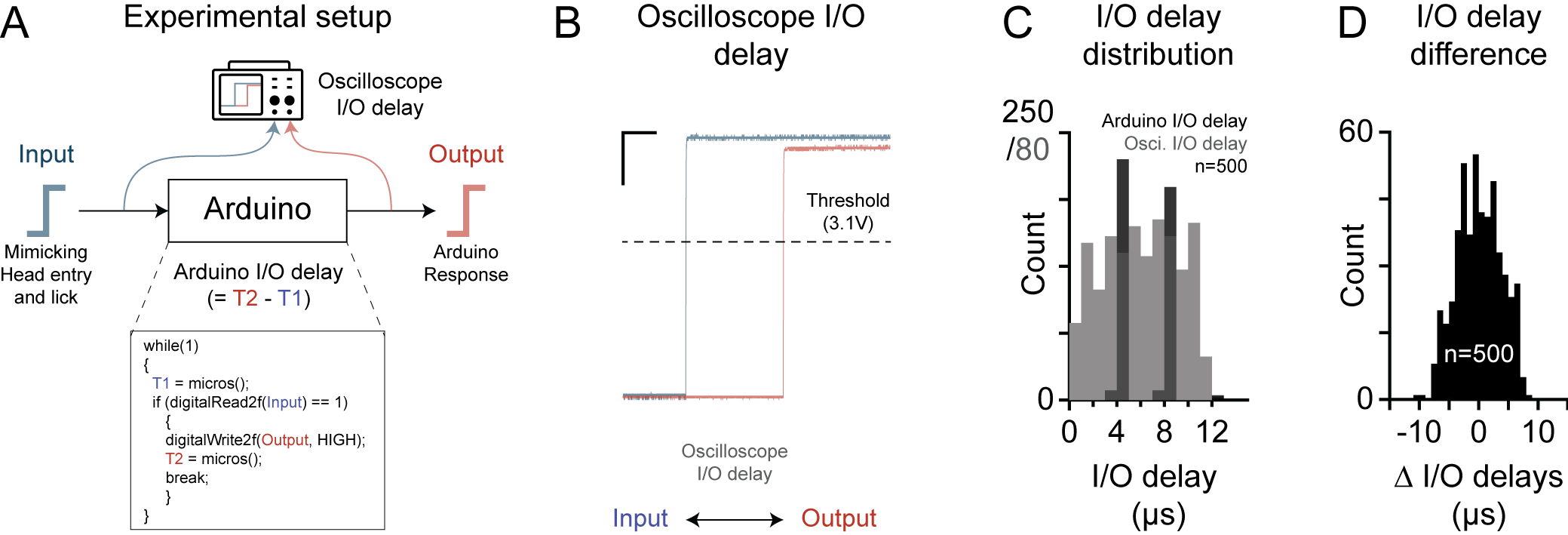

Supplement: Figure 1-1 — Evaluation of the input/output reactivity of FreiBox. A, B, Measuring behavior requires integration and control of a myriad of different instruments in response to animal behavior by respecting a submillisecond timescale resolution. For example, the interlick interval is distributed at ∼100 ms (Fig. 2D), meaning that an Arduino controller has to integrate external information very quickly to measure animal behavior. To quantify the speed of FreiBox to integrate, respond, and time stamp such digital inputs, we conducted an experiment to measure the response reactivity (“I/O delay”) of an Arduino Mega to respond to an incoming TTL input. A, Experimental setup and Arduino code used to measure the I/O delay of an Arduino Mega (“Output”) receiving an incoming TTL input (“Input”) driven by an experimenter controlling a 5V manual-push button. Arduino Mega was programmed to register the time stamp of the Input TTL (T1), detected with the Arduino library “DIO2,” and the time stamp of the TTL output (T2) right after its emission. In parallel, an oscilloscope (model DSO-1204e, Voltcraft) connected to a computer (DSO3104 software) recorded both input and output TTL to quantify the I/O delay at a fast sampling (500 kHz). B, Example of the TTL traces recorded with an oscilloscope (sampling rate, 500 kHz). Calibration: 5 μs, 1 V. The oscilloscope I/O delay was obtained offline by measuring the delay between the input and output TTLs when their voltages were crossing 3.1 V (dashed line). C, D, In this experiment, we used 2 different boxes and mimicked 250 TTL inputs in each box by pressing a push button (n = 500 total trials). For these boxes, the I/O delays recorded with the Arduino (Box1 vs Box2: 6.03 × 10^–6^ ± 1.35 × 10–7 s vs 5.86 × 10^–6^ ± 1.28 × 10–7 s; n = 250 vs 250; Mann–Whitney rank-sum test: t = 64066.5, p = 0.359) and the oscilloscope (Box1 vs Box2: 5.90 × 10^–6^ ± 2.01 × 10–7 s vs 6.13 × 10^–6^ ± 1.94 × 10–7 s; n = 250 vs 250; Mann–Whitney rank-sum test: t = 61359, p = 0.433 [file enu-eN-OTM-0469-22-s07.tif]

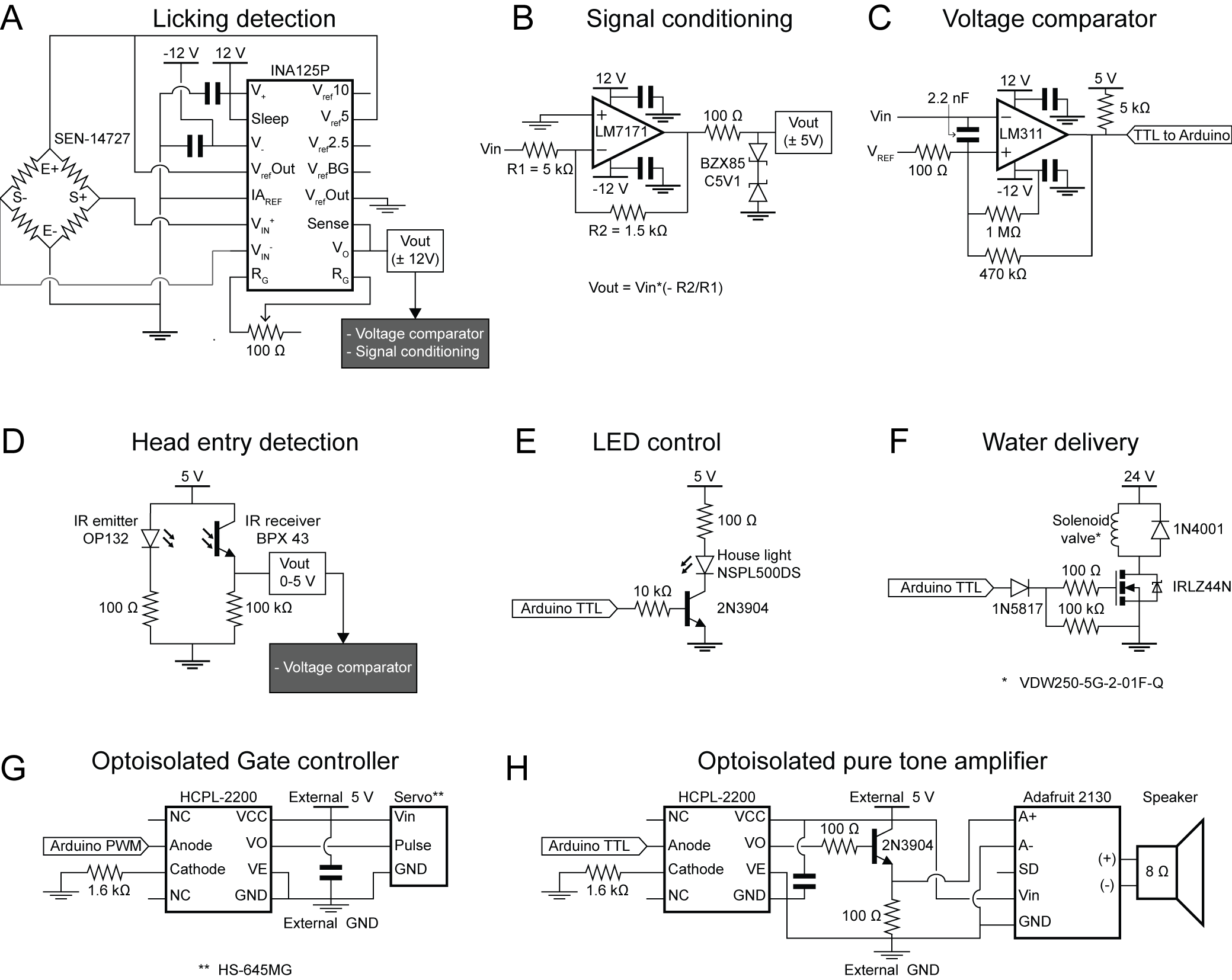

Supplement: Figure 1-2 — Electronic circuits of the FreiBox modules. A, B, Amplification (A) and conditioning (B) circuit of the SG signal. The signal conditioning circuit is used to divide the voltage of the amplified signal (±12V) in a range that is acceptable for recording with most of DAC board (±5V). Note that the 100 Ω trimmer in A is used to adjust the sensitivity of the instrumental amplifier. C, Voltage comparator circuit used to generate a TTL when the input voltage (Vin) exceeds the value of a reference voltage (Vref). This circuit is used to detect both individual licks, and the IR beam breaking induced by a head entry in the NP or LC. The Vref is controlled by a precise potentiometer (10 kΩ; tolerance, 5%; 10 turns) to fine-tune the licking detection or by a trimmer for head entry detection. D, Electronic circuit of the head entry detection system. The generated signal (Vout) is sent to the voltage comparator circuit described in C to generate TTL signal during a NP. E–H, Electronic circuits of the Lighthouse LED Controller (E), water delivery system (F), the optoisolated gate controller (G), and the optoisolated auditory cure amplifier (H). Optoisolators are used to reduce the electrical noise induced by the motor in the FreiBox circuit and in the speaker. Download Figure 1-2, TIF file. [file enu-eN-OTM-0469-22-s06.tif]

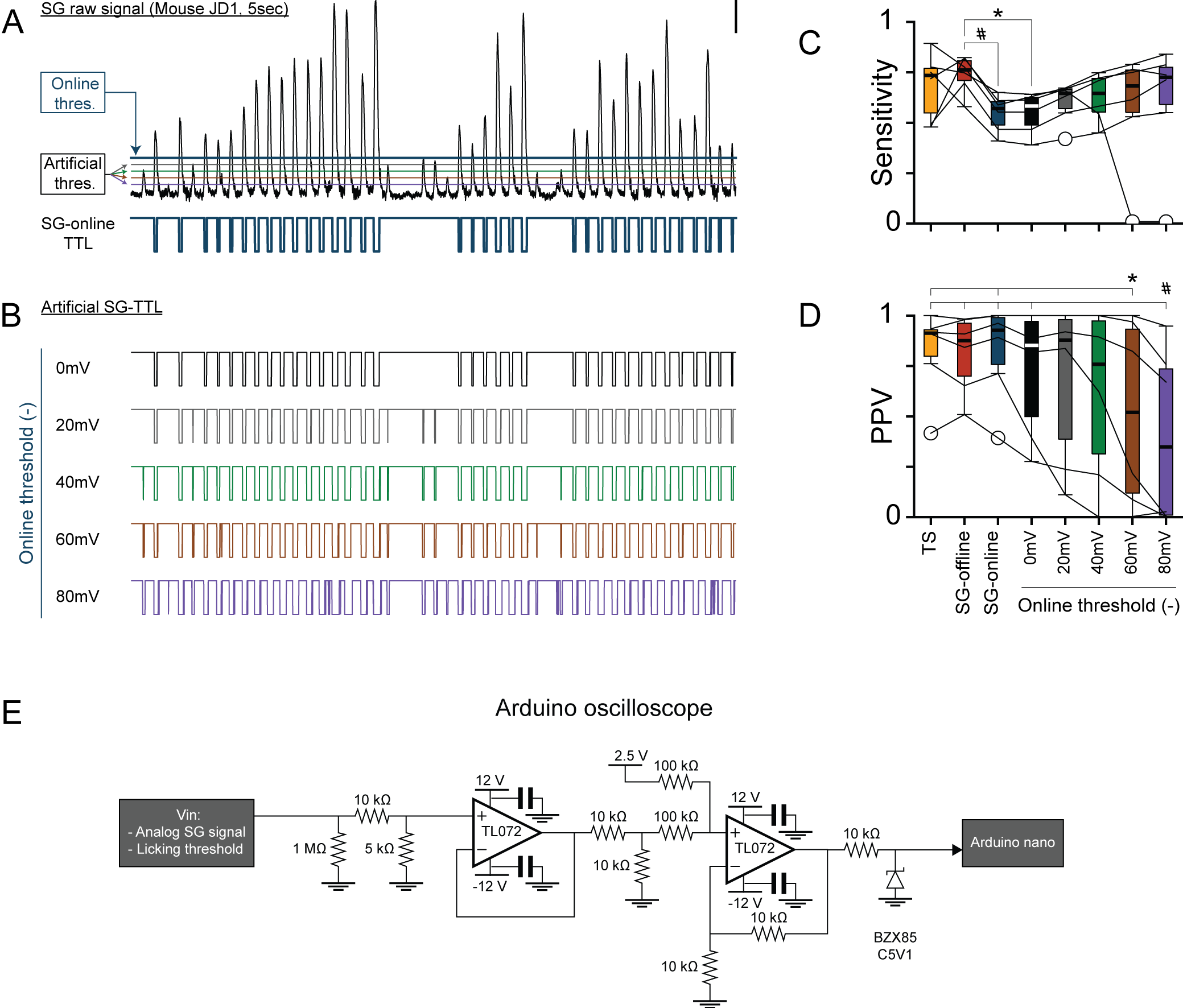

Supplement: Figure 2-1 — Improving online detection of the SG lickometer. A, B, A 5 s example of the SG raw signal (A; calibration, 0.1 V) and its online detection signal (B; SG-online TTL) recorded from another mouse (#JD1) than in Figure 2C. The SG-online TTL is generated when the SGs crosses an online reference voltage threshold set 100 mV above the baseline (“Online thres.”). By decreasing artificially this reference voltage (A, Artificial thresh.), we generated multiple artificial SG-TTL traces (B) to evaluate the effect of the voltage threshold on the sensitivity (C) and the PPV (D). C, Box-and-whisker plots comparing the sensitivity of SG lickometer and of the artificial SG-TTL traces as described in B (n = 6; Friedman repeated-measures ANOVA on ranks: χ2 = 21.643, df = 7, p = 0.003; TS vs SG-offline vs SG-online vs 0 mV vs 20 mV vs 40 mV vs 60 mV; 0.685 ± 0.0676 vs 0.740 ± 0.0374 vs 0.546 ± 0.0372 vs 0.549 ± 0.0413 vs 0.600 ± 0.0406 vs 0.626 ± 0.0486 vs 0.577 ± 0.121 vs 0.606 ± 0.126, respectively; describe data: mean ± SEM). *#Tukey’s post hoc test, q = 5, p < 0.05. D, Box-and-whisker plots comparing the PPV of SG lickometer and of the artificial SG-TTL traces as described in B (one-way repeated-measures ANOVA: n = 6, F = 5.523, p < 0.001; TS vs SG-offline vs SG-online vs 0 mV vs 20 mV vs 40 mV vs 60 mV; 0.823 ± 0.0873 vs 0.813 ± 0.0785 vs 0.827 ± 0.0972 vs 0.729 ± 0.129 vs 0.684 ± 0.164 vs 0.621 ± 0.175 vs 0.517 ± 0.189 vs 0.402 ± 0.178, respectively; describe data: mean ± SEM. #Tukey’s post hoc test; 80 mV vs TS, 80 mV vs SG-Offline, 80 mV vs SG-Online, 80 mV vs 0 mV; q = 86.365, 86.205, 86.417, 84.941, respectively; p = 0.002, 0.002, 0.002, 0.026 respectively. *Tukey’s post hoc test; 60 mV versus TS and 60 mV versus SG Online; q = 84.630 and 84.683, respectively; p = 0.044 and 0.04, respectively. E, Electronic circuit of the Arduino oscilloscope for online monitoring of the SG signals and their thresholds. Design modified from https://create.arduino.cc/projecthub/aimukhin/advanc [file enu-eN-OTM-0469-22-s05.tif]

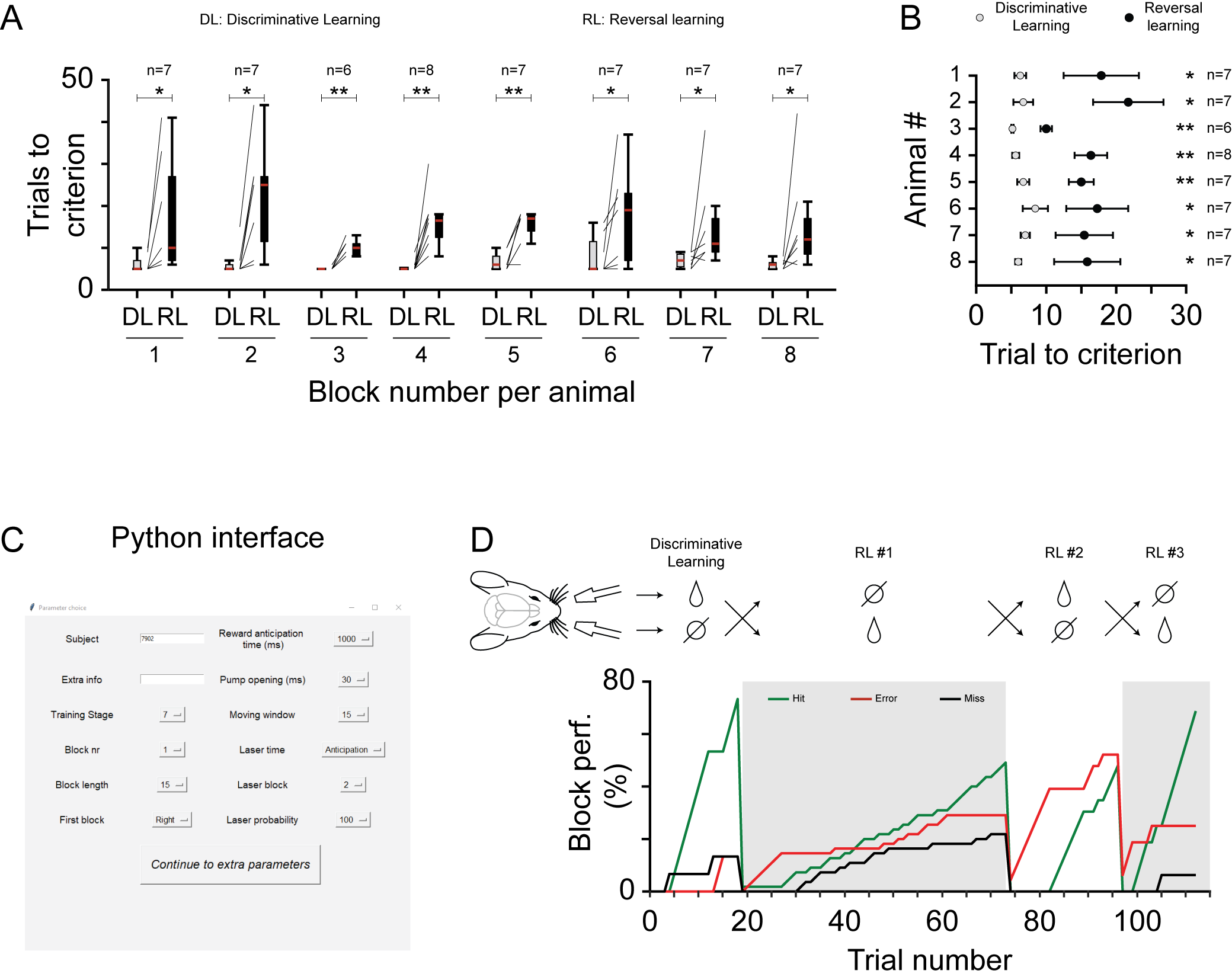

Supplement: Figure 3-1 — Individual behavioral data and serial reversal learning setting. A, B, Number (A) and average (B; mean ± SEM) of trials to reach the criterion of each individual mouse used in Figure 4. For mice 1 (n = 7, t = –2.492, p = 0.047), 2 (n = 7, t = –3.739, p = 0.01), 3 (n = 6, t = –6.100, p = 0.002), 4 (n = 8, t = –4.633, p = 0.002), and 5 (n = 8, t = –4.757, p = 0.003), a paired t test was performed to compare the numbers of trials to criterion during DL and RL blocks. In contrast, Wilcoxon signed-rank tests were performed for the mice 6 (n = 7, z = 2.201, p = 0.031), 7 (n = 7, z = 2.201, p = 0.031), and 8 (n = 7, z = 2.197, p = 0.031). * and ** show significance at the risk α = 0.05 and 0.01, respectively. C, D, By changing the number of blocks in the GUI (C), we are able to control a serial intrasession RL in well trained Thy1-GCamP6f mice as shown in D. OFC GCamP6f expression as well as Ca2+ imaging recorded during the same behavioral session are shown in Extended Data Figure 4-2. Download Figure 3-1, TIF file. [file enu-eN-OTM-0469-22-s04.tif]

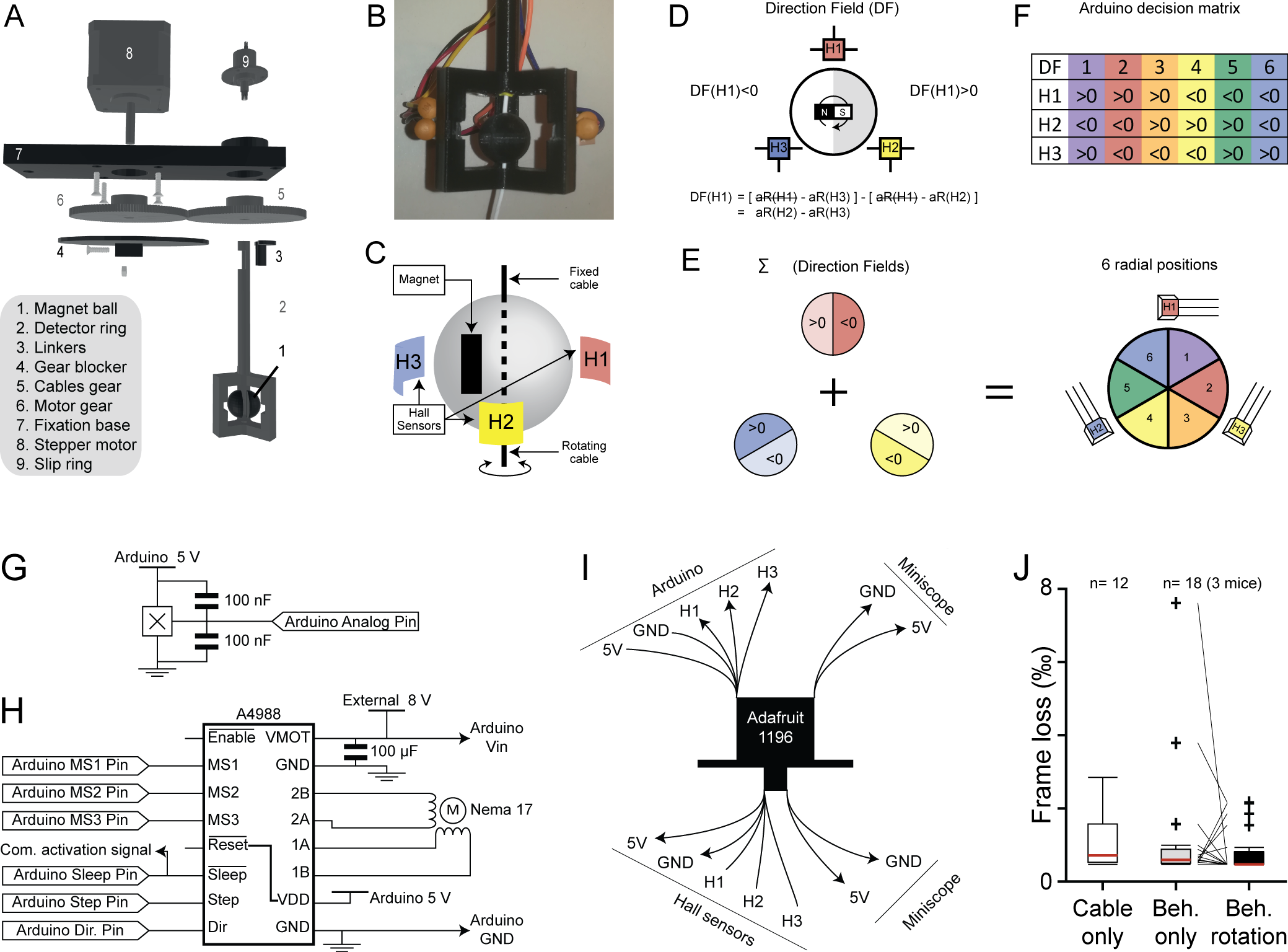

Supplement: Figure 4-1 — The FreiBox commutator. A–C, Neurophysiological recordings in freely moving animals require untangling of the Miniscope coaxial cables connected to the freely moving implanted animal. To solve that problem, previous designs developed a well established method to detect the rotation of a cable implant by tracking the relative position of a magnet to a sensor called a Hall sensor (Fee and Leonardo, 2001; Liberti et al., 2017; Barbera et al., 2020). By modifying a commutator from the Gardner laboratory (Liberti et al., 2017), we designed a low-cost (111 €) open-source commutator as described in the exploded view (A). By adding a magnetic ring rotary encoder (B), we improved the detection of the cable position and constrained the cable movement. This design offers the advantage of maintaining the magnet close to the detector ring even when the connected mouse moves in a spacious environment. The schematic representation of the ring commutator (C) shows that a magnet glued to the Miniscope coaxial cable can rotate along the cable axis among the 3 fixed Hall sensors arranged in a circle around the cable (H1, H2, and H3). D, To track the position of the magnet into the ring detector, the direction field (DF) of each Hall sensor is calculated as described by an Arduino using the function analogRead (“aR”). E, F, By combining the direction fields of the 3 Hall sensors (E), it is possible to define the orientation of the magnet in the field forms by the 3 Hall sensors (among 6 radial positions) and adjust the cable position on the basis of a decision matrix (F). G–I, To build the FreiBox commutator, the 3 Hall sensors (G) and a motor driver card (H) are connected to the step motor and the Arduino; the signals from the Miniscope and the Hall sensors are relayed via a slip ring (I). J, To test whether the commutator rotation induces additional data loss during Miniscope recordings, we compared the percentage of frame loss when the Miniscope was connected to the DAC board with a [file enu-eN-OTM-0469-22-s03.tif]

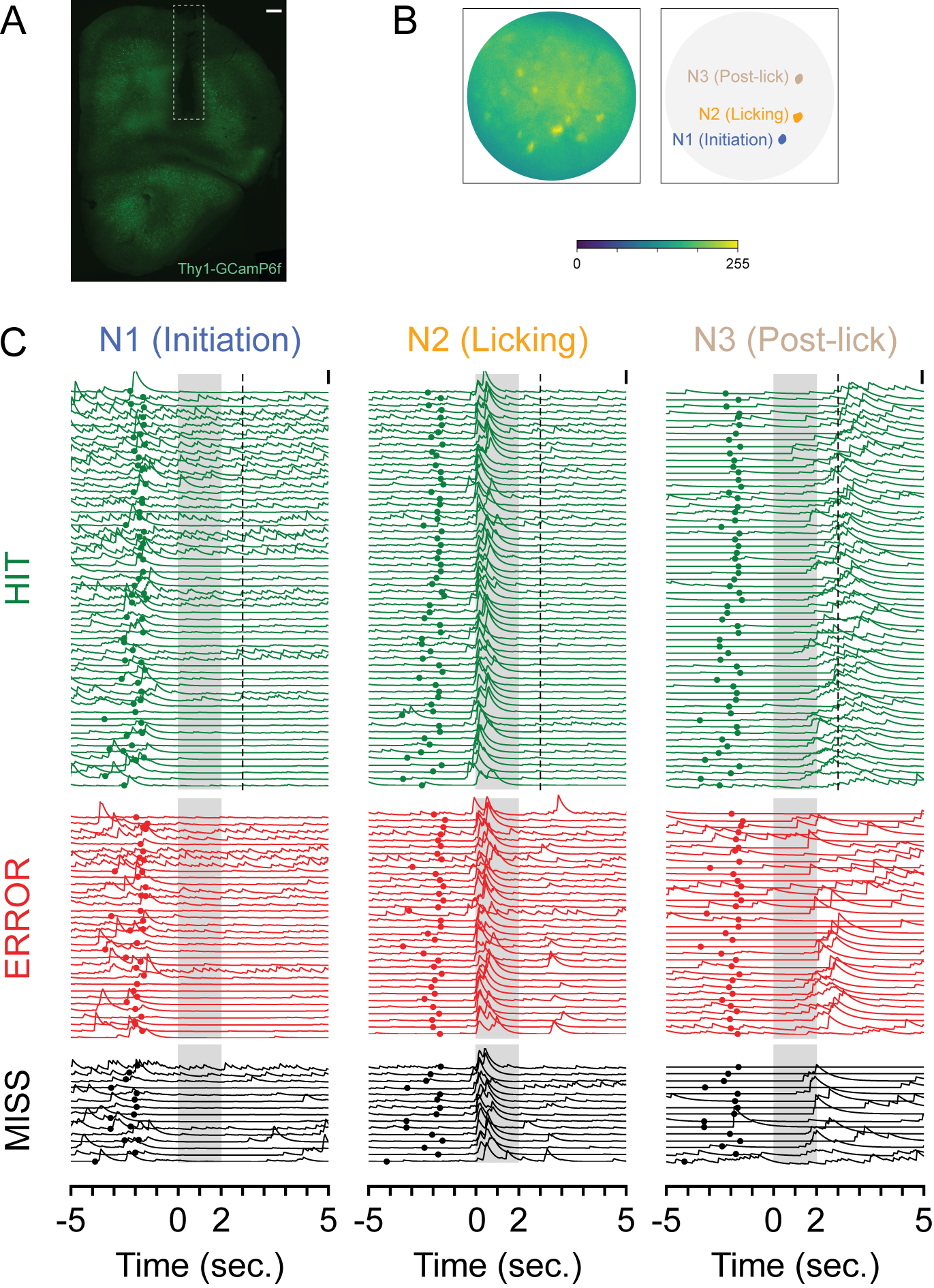

Supplement: Figure 4-2 — OFC calcium imaging during intrasession RL. A, Thy1-GCaMP6f expression and GRIN lens track in OFC. Scale bar, 250 μm. B, C, Examples of three modulated neurons (B) and their calcium traces displayed as column (C) aligned to the head entry in the LC (0 s) and split between hit, error, and miss trials during the same behavioral session as shown in Extended Data Figure 3-1D. The maximum projection picture (B, left) was obtained on the first 10,000 frames of the recording session. The spatial footprints (B, right) and the normalized calcium traces (C) were obtained with the open-source pipeline CalmAn (gSig/gSiz: 3/13). The calcium traces represent the normalized denoised temporal traces (CalmAn parameter C; scales, 5 normalized df) and were colored in accordance with their spatial footprints shown in B, right. Normalized calcium traces were aligned to the beginning of the LR windows (shaded in gray), sorted by trial type (hit, error, or miss) and their trial number (shown here in an ascending order). The colored dots indicate the nose poke time stamps. For the hit trials, the dashed lines indicate the end of the anticipatory response windows, immediately followed by a water reward delivery. Download Figure 4-2, TIF file. [file enu-eN-OTM-0469-22-s02.tif]
